# Predictability and Agency Shape Self-Touch Perception

**DOI:** 10.1525/collabra.160692

**Published:** 2026-05-04

**Authors:** Adam Enmalm, Eleonora Vagnoni, Patrick Falk, Aikaterini Fotopoulou, Rebecca Boehme

**Affiliations:** 1 Center for Social and Affective Neuroscience, Linköping University, Linköping, Sweden; 2 Psychological and Social Sciences, John Cabot University, Rome, Italy; 3 School of Bioscience, Högskolan i Skövde, Skövde, Sweden; 4 Research Department of Clinical, Educational and Health Psychology, University College London, London, UK

**Keywords:** Social softness illusion, self-touch, affective touch, CT, agency

## Abstract

Self-touch and interpersonal touch feel very different—self-touch sensations are attenuated. The distinction between self- and not-self not only influences properties such as intensity, but also qualitative aspects of touch. This is highlighted by the social softness illusion, wherein another person’s skin is perceived as softer and smoother than the own skin. The mechanisms underlying these qualitative differences of self- vs social touch remain unclear. Across four experiments, we explored such qualitative aspects of touch sensation in eighty-two healthy females. We investigated both the social softness illusion and self-touch across multiple body parts. We found that the social softness illusion only occurs under a specific condition: when the palm was touching the forearm. When the forearm instead touched the palm, a self-softness illusion emerged. For self-touch, we found differences between forearm touching palm and palm touching forearm, which were not directly linked to the movements of the palm or forearm. We also found an interaction between movement and velocity for pleasantness, where velocity mattered more during external than self-generated movements. Agency too influenced the qualitative aspects, particularly interacting with the velocity. We corroborate earlier studies showing a preference towards social touch for social interactions and the suggested soothing mechanism of self-touch. The hedonic benefits of self and social touch emerge for different reasons, therefore comparing these competing systems is complex. When comparing the qualitative aspects of social and self-touch, one must consider these two complimentary functions, with potentially different underlying psychological and neurophysiological substrates.

## 1 Introduction

Touch has a central function in human social behavior, and is important throughout development for attachment, social interactions and communication (Cascio et al., 2019). Interpersonal touch not only carries hedonic benefits (Mazza et al., 2023), but is also shown to affect physiology, with lower heart rate and increased heart rate variability (Candia-Rivera et al., 2025; Triscoli et al., 2017). A physiological modulation of touch has been found even in preterm babies (Püschel et al., 2022).

The perception of social touch is contextual and depends on many factors, such as the relationship to the toucher, cultural factors, and more (Schirmer et al., 2023; Suvilehto et al., 2019). Touch carries both discriminative and affective components, that are distinguishable (Olausson et al., 2002). These characteristics of touch also relate to processing in different brain regions, namely the somatosensory cortex for the component, and the insula for the affective component (McGlone et al., 2012; Olausson et al., 2008). Slow conducting C-tactile (CT) receptors located primarily in hairy skin (Olausson et al., 2010) show preferential activation to soft, slow stroking at skin temperature and their activation relates specifically to hedonic aspects of touch (Ackerley, Backlund Wasling, et al., 2014; Löken et al., 2009). Sporadic innervation of CT-afferents has been reported for glabrous skin and the glabrous-hairy skin border at roughly one seventh the innervation density (Watkins et al., 2021), although this has not been replicated.

Most research on touch has focused on being touched, however providing touch also carries hedonic benefits (Gentsch et al., 2015). A compelling example of the hedonic benefits of providing touch is the social softness illusion (SSI) (Gentsch et al., 2015) postulating that another person’s skin is perceived as softer than the own skin. The illusion seems to be driven specifically by the skin-to-skin contact, as introducing a piece of cloth covering the skin abolished the illusion. Interestingly, the SSI shows a similar tuning to velocity as the CT afferents, with the highest illusory strength at 3 cm/s and 10 cm/s. Further, the illusion seems to be driven by active movements – the illusion does not appear during static touch nor passive touch (i.e. hand being moved externally). The SSI might therefore occur specifically when the toucher employs a type of touch CT-afferents respond to, and which would with high probability be perceived as pleasant or desired by the recipient.

However, this cannot be the only driving factor, as the same type of touch to one’s own arm would also activate CT-fibers and would therefore be considered affective. The contextual knowledge that another person is being touched must contribute to the SSI. Contrary to interpersonal touch, self-touch has both a touching and touched sensory component (Katz & Krueger, 2013). Counterintuitively, the experience is not enhanced during self-touch. Instead, it is attenuated. With matched intensities, the self-touch experience is phenomenologically weaker (S. J. Blakemore et al., 1998; Kilteni, 2023; Shergill et al., 2005; Weiskrantz et al., 1971), potentially due to the high level of predictability of self-touch. Already Helmholtz suggested that when a movement is executed, an efference copy of the motor information is used to create a prediction of the sensory outcome of the movement (von Helmholtz, 1867). If the predicted outcome matches the sensory outcome, the resulting sensation is attenuated (S. J. Blakemore et al., 1998, 2000; Boehme et al., 2019; Boehme, 2025; Boehme & Olausson, 2022; Brown et al., 2013; Desantis et al., 2012; Gentsch & Schütz-Bosbach, 2011; Shergill et al., 2005; Weiss et al., 2011). However, this prediction seems to be adaptable, at least in the temporal domain (Kilteni et al., 2019; Kilteni & Ehrsson, 2024). Attenuation decreases as spatial discrepancy increases (Kilteni et al., 2018). The degree of attenuation has been linked to connectivity between the cerebellum and secondary somatosensory cortex (Kilteni & Ehrsson, 2020). Interestingly, imagined movements (Kilteni et al., 2018) and incorrect action attribution also cause attenuation (Burin et al., 2018), suggesting that not only the simple motor command causes the effect. However, while the sensation of self-touch is attenuated, it is not abolished, in line with the idea that self-touch does serve a function (Boehme & Olausson, 2022), potentially self-soothing (Uvnäs-Moberg et al., 2015), emotion and attention regulation (Spille et al., 2022) as well as contributing to the development of the bodily self model.

The self-other distinction of touch may be heavily influenced by the attenuation of self-touch, and the differences between self-touch and interpersonal touch for discriminative aspects is well described. As shown with the SSI being abolished with the introduction of cloth (during which attenuation should still be present), the factors influencing how self-touch differs to interpersonal touch in the qualitative and affective aspects are not well known. It remains unclear how the bottom-up sensory input interacts with potential top-down modulations, predictions, and contextual factors, as these are known to influence the experience of touch (Cascio et al., 2019; Suvilehto et al., 2019). Across four experiments, we examined factors known to influence qualitative aspects of touch. We examined if and how the SSI varies across body parts with varying CT-innervations, both regarding the delivering and receiving body parts (experiment 1). We further examined how movements interacted with these body parts to investigate the dual-touch during self-touch (experiment 2). We examined how active and passive movements interacted with velocity in shaping qualitative experiences of soft stroking with a brush (experiment 3). Finally, we assessed whether agency rather than movement influences touch and interacts with velocity to shape the tactile experience (experiment 4).

## 2 Experiment 1

### 2.1 Problem Formulation

Experiment 1 aimed to explore the role of the CT-system in the SSI. Since the SSI showed a similar velocity tuning as the CT-system and occurred only when the target area was heavy in CT-innervation, it was suggested that it might occur specifically when activating CTs in the touch-recipient (Gentsch et al., 2015). To explore this, a similar setup to the original SSI study was employed, wherein the participant comparatively touched themselves and another person on palm and forearm. However, we extended the design to also include the forearm (hairy skin) as a delivering site in addition to the palm (glabrous skin). We hypothesized the SSI to emerge when touching palm-on-forearm (i.e. replicating the original SSI study) as well as when touching forearm-on-forearm, but not when touching with palm-on-palm or forearm-on-palm (given the lower innervation of CTs on glabrous skin).

### 2.2 Methods

#### 2.2.1 Participants

Thirty naïve female participants were recruited for experiment 1 (age = 22.8 ± 3.24, range 19-31). Participants were not familiar with each other prior to study enrollment, and verbal communication between participants was restricted to limit the influence of other social factors. Participants were right-handed (n = 29) or ambidextrous (n = 1), as assessed by the EHI (Oldfield, 1971). Participants were recruited from the UCL Psychology subject pool. While no main effect of gender was expected, only females were included to minimize potential mixed gender interactions and the variance in physical skin properties (i.e., hair density, see for example Bhushan et al. (2005)). To further limit the influence of variance in skin properties, participants were assigned into pairs, alternating between being the active and passive participant. Exclusion criteria included any history of psychiatric or neurological disorders, as well as any skin conditions affecting the touched areas, such as scarring or tattoos. This was determined via self-report. The study was approved by the institutional ethical committee Department of Clinical, Educational and Health Psychology, UCL), and all participants gave their written informed consent prior to experiment start.

#### 2.2.2 Setup

**Figure 1:**
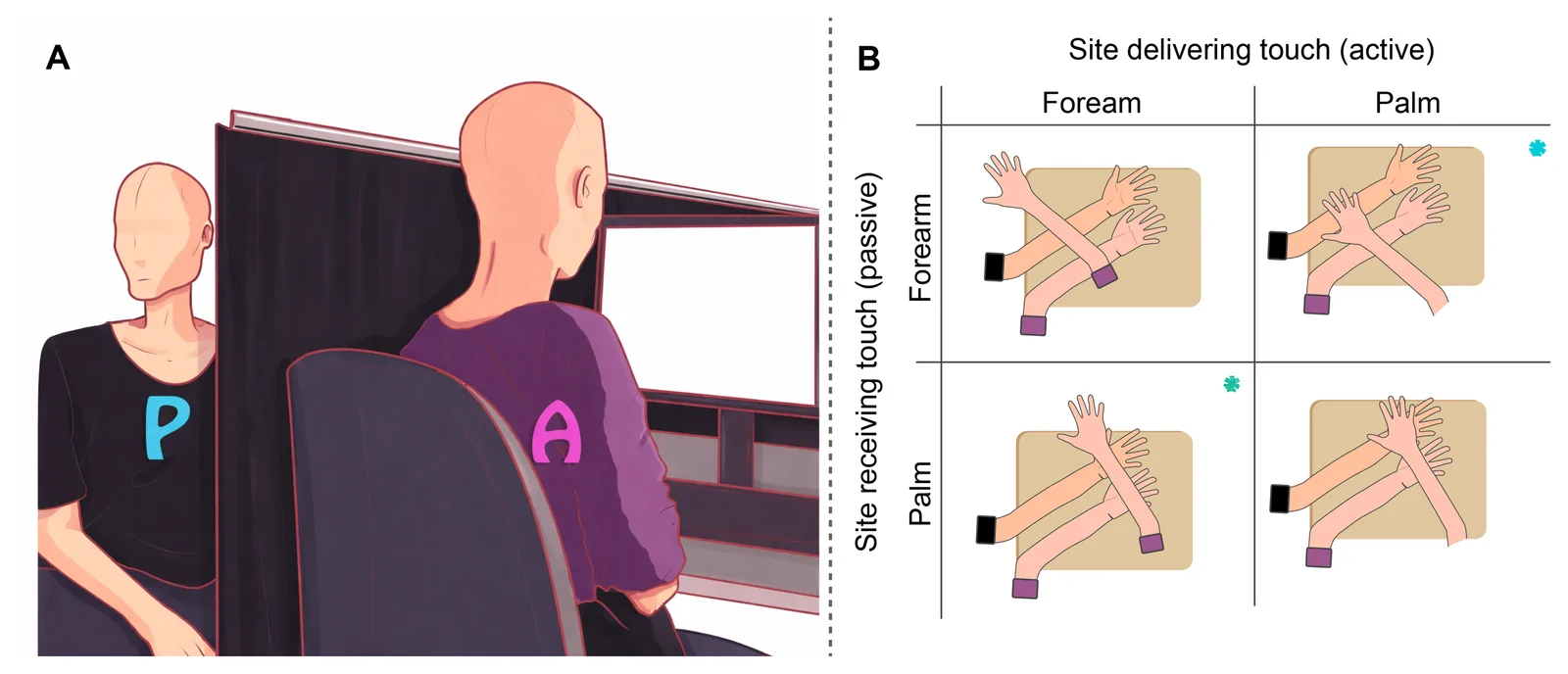
A) Setup in experiment 1. The active participant (A) was facing a computer screen on which instructions were shown and ratings provided. The passive participant (P) sat on the other side of a curtain with their left arm accessible for the active participant to touch. The active participant placed their arm next to the passive participant’s arm, shown in panel B. B) The four types of touch conditions in experiment 1. All touch conditions consisted of touching their own (purple shirt) forearm/palm followed by the other person’s (black shirt) forearm/palm. The order of touch (self vs other) was counterbalanced within subjects. Experiment 1 focused primarily on non-matched body parts, highlighted with a star. Forearm-on-palm in teal, palm-on-forearm in blue.

Participants were seated next to each other facing opposite directions (Figure 1A). The active participant, responsible for delivering the touch, was facing a computer screen where task instructions were provided. The passive participant sat left of the active participant, with their back facing the table. The participants were separated by a curtain to minimize visual input. Both participants rested their forearms on the table in a parallel position, angled approximately 45° relative to the active participant. To standardize the area of contact, a 9-by-4 cm area was marked on the volar side of each participant’s forearm and palm, both of which were facing upwards. The active participant was instructed to fixate on the computer screen where a “+” was presented.

The experiment consisted of four conditions in a 2-by-2 within-subject design. These conditions included forearm touching forearm, forearm touching palm, palm touching forearm, and palm touching palm (Figure 1B). The active participant touched their own hand or forearm followed by the corresponding body part in the passive participant, and vice versa, touch order counterbalanced within participant. Instructions for each condition and responses were recorded using Presentation (Neurobehavioral Systems) via the screen placed before the active participant.

#### 2.2.3 Procedure

The task was divided into two blocks, the order of which was randomized and counterbalanced across participants. In one block, the active participant applied touch using their fingertips (palm), and in the other, using their volar forearm. This was counterbalanced across participants. Touch occurred to either the palm or forearm, alternating within blocks. The active participant touched either themselves followed by touching the collaborator participant, or in the inverted order (alternating within blocks). This was to avoid any anchoring of preferences. Touch occurred on both sites but were always paired (i.e. comparative ratings were always within location). After each pair of touches (i.e. self and other), the active participant provided comparative ratings of the two sites touched across three dimensions: softness, smoothness, and comfort. The presentation order of these ratings was randomized within participants. Each dimension was rated on a 200mm visual-analog scale (VAS). The anchors were labeled “Other Skin” (left) and “Own Skin” (right), with the midline representing no preference. A total of 8 ratings per dimension were completed per block. Upon completion of the first block, a short break was provided, after which the second block began, mirroring the first in structure but switching the touching body part. As such, four touch “models” occurred, palm-palm, palm-forearm, forearm-palm, and forearm-forearm. Once both blocks were completed, the roles of the participants (active/passive) were swapped, and the entire procedure was repeated.

#### 2.2.4 Data analysis

All data was analyzed using JASP version 0.19.3 (JASP Team). The VAS positioning was converted into numerical values ranging from -100 to +100, with negative values indicating a preference for the other’s skin and positive values indicating a preference for one’s own skin. The three dimensions (i.e., softness, smoothness, and comfort) were analyzed separately. A linear mixed model with the Satterthwaite method was done for all rating scales. Fixed effects were touch interaction (i.e. palm-palm, palm-forearm, forearm-palm, and forearm-forearm), and random grouping effects were participant and pair (i.e. touching dyad). The formula as such read **rating ~ touch interaction + (1** | **dyad) + (1** | **subject)**. To appropriately test whether participants exhibited a significant preference for either self- or other-touch in each condition, post-hoc marginal means were tested against zero, corrected for multiple comparisons using the Bonferroni-Holms correction. Effect sizes of each model are reported using Cohen’s f^2^ and standardized fixed-effect estimates are reported for the follow-up tests.

### 2.3 Results

#### 2.3.1 Softness

A significant effect of touching condition was found, F (3, 19.14) = 9.287, p < 0.001, f^2^ = 0.593. Post-hoc tests indicated that palm-forearm showed preference to “other”, z = -3.889, p < 0.001, fixed-effect estimate = -18.812. Conversely, when using the forearm to touch the palm, participants perceived their own skin as softer, z = 3.611, p < 0.001, fixed-effect estimate = 18.171 (fig. 3a). No significant preference of skin (self vs. other) was found for the touch interactions forearm-to-forearm, *z =* 0.394, p = 0.693, fixed-effect estimate = 1.029, or palm-to-palm, z = 0.183, p = 0.855, fixed-effect estimate = 0.838.

#### 2.3.2 Smoothness

For smoothness, the same effects were found, F (3, 19.25) = 7.988, p = 0.001, f^2^ = 0.555 (fig. 3a). When touching the other’s forearm with the palm, a preference of other was found, z = -3.508, p < 0.001, fixed-effect estimate = -15.873. When the forearm was used to touch the other’s palm, a preference of self was found, z = 3.072, p = 0.002, fixed-effect estimate = 18.944. No significant preference of skin (self vs. other) was found for the touch interactions fore-arm-to-forearm, z = 0.029, p = 0.977, fixed-effect estimate = 0.880, or palm-to-palm, z = -0.855, p = 0.392, fixed-effect estimate = -0.660.

#### 2.3.3 Pleasantness

For all touch interactions, the linear mixed model was not significant, F (3, 19.20) = 2.371, p = 0.102, f^2^ = 0.270, indicating no main effect of condition on pleasantness ratings. Nonetheless, follow-up tests were done to investigate preferences. The own skin was rated as more pleasant to touch in all conditions (fig. 3a). This effect was significant for forearm-to-forearm, z = 2.675, *p* = 0.007, fixed-effect estimate = 7.269, forearm-to-palm, z = 3.676, *p* < 0.001, fixed-effect estimate = 15.540, and palm-to-forearm, z = 2.447, *p* = 0.014, fixed-effect estimate = -3.415a. Palm-to-palm was descriptively similar but did not reach significance, z = 1.842, *p* = 0.065, fixed-effect estimate = 1.719.

### 2.4 Interim Discussion Experiment 1

Experiment 1 investigated the SSI across different body parts. We manipulated the delivering and a receiving body part, and by extension also the skin type, glabrous (CT-) or hairy skin (CT+). Consistent with the original SSI study (Gentsch et al., 2015), participants perceived another person’s skin as softer and smoother than their own when touching the forearm with their palm, and reported no preference when touching with the palm on the palm. Contrary to our hypothesis, we observed an inverse SSI (or a self-softness illusion) in which participants perceived their own skin as softer, when the body parts were reversed, i.e., when the forearm was touching the palm. Forearm-to-forearm did not show significant preference. Together, this shows that the SSI is not dependent solely on the social interaction or skin type, but rather on the specific combination of involved body-parts / skin-types.

During self-touch, dual touch occurs, as sensory information from the experience is provided from both touching and touched body part. During interpersonal social touch, sensory information is only provided from one part (Katz & Krueger, 2013). This is in addition to the attenuation, i.e. reduced perceptual intensity of self-touch (S.-J. Blakemore et al., 1999; Kilteni & Ehrsson, 2020). While the conditions under which attenuation occur are relatively well known, how the dual touch inputs are combined and considered during self-touch remain a contemporary topic. A recent study showed that self-touch is perceived and processed different even when the constituents are matching and mimicking the dual touch, i.e. giving and receiving touch simultaneously (Enmalm et al., 2025) This difference seems to be driven primarily by the touched body part. It is therefore possible that participants in the current experiment rated the qualitative aspects of touch primarily regarding their stationary touched body part. When moving the palm over the own forearm, the own fore<arm feels the texture of the palm, i.e. the perception of the stationary body part trumps that of the moving body part, thereby making the touch less smooth and soft compared to touching the other person’s forearm, where only the soft/smooth forearm is felt (by the own touching palm). When touching forearm on palm, the inverse occurs: during self-touch the stationary palm feels primarily the smooth forearm, whereas for the interpersonal touch the moving forearm feels the rough palm.

Finally, we replicated the original SSI, with a preference of touching the own skin rather than the other persons skin when rating pleasantness. Self-touch is typically regarded as less pleasant than being touched by someone else (S. J. Blakemore et al., 2000; Enmalm et al., 2025). While social touch is often more pleasurable when received rather than given (Enmalm et al., 2025; Triscoli et al., 2017), the act of giving touch still holds affective value (Enmalm et al., 2025; Gentsch et al., 2015; Maister et al., 2020; Triscoli et al., 2017). The context of our study (i.e., touching between unfamiliar strangers) may help explain discrepancies with earlier work, as touch between two strangers could dampen the hedonic experience of both giving and receiving social touch.

## 3 Experiment 2

### 3.1 Problem Formulation

Experiment 2 tried to clarify the role of movement and body part during the dual touch that occurs during self-touch. A similar setup to that of experiment 1 was used, however employing only self-touch. We now introduced the additional factor of movement. The movement could either originate from the top “delivering” body part (palm/forearm), or the bottom “receiving” body part, to investigate if ratings differed when the body parts alternated in being active or passive.

Given the discrepancy of preferences for forearm-palm and palm-forearm observed in experiment 1, analysis of experiment 2 focused specifically on these comparisons. All other site and movement combinations are available in the supplement. We hypothesized a similar pattern to that of experiment 1 – higher ratings of softness and smoothness when touching forearm-on-palm rather compared to palm-on-forearm. In line with the dual touch premise, we also hypothesized ratings to interact with movement, particularly that ratings would be higher when the forearm moved, regardless of site configuration (forearm as top body part, or as bottom body part), as perception of the palm feeling the softness of the forearm would then dominate the rating.

### 3.2 Methods

#### 3.2.1 Participants

Thirty female participants were recruited (age = 21.2 ±2.7, range 18-28). This sample was independent from experiment 1 and had no prior exposure to any of the tasks. Participants were right-handed (n = 29), or ambidextrous (n = 1) assessed by the EHI (Oldfield, 1971). The same inclusion and exclusion criteria used in experiment 1 was applied, and all participants provided written consent prior to experiment start.

#### 3.2.2 Setup

Participants were seated in front of a computer screen and asked to fixate on a “+” presented on the screen. The left arm was placed palm facing upwards on a moveable platform with a skateboard attached underneath it. The skateboard platform was placed in a wooden frame allowing it to move 9 cm back and forth, but preventing sideways movement. Participants had a 9x4 cm area marked in a proximal-distal orientation along their left forearm or palm (n = 15 per area). As in experiment 1, touch was a soft slow stroke, targeted at CT-optimal velocity (3 cm/s). When the movement originated from the top body part, the participant moved their right arm along the marked zone (fore-arm/palm) in a distal to proximal direction. When movement originated from the bottom body part, the right arm instead remained stationary, and the participant moved their left arm in a proximal-to-distal direction. To ensure appropriate tactile velocity, the movement was timed to take 3 seconds, resulting in a velocity of 3 cm/s (total displacement of 9 cm).

**Figure 2:**
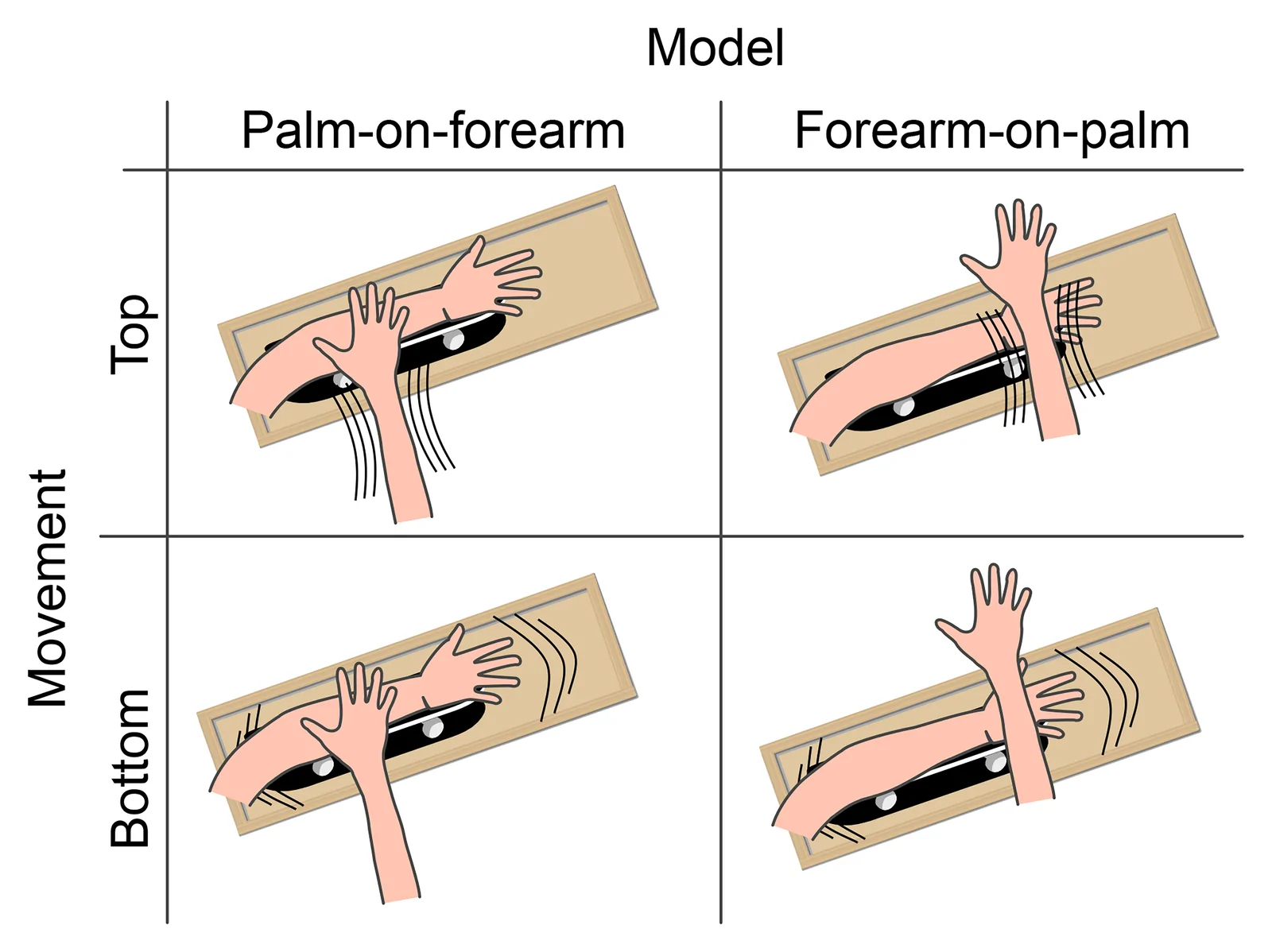
Setup of Experiment 2. Touch could either be on the palm or forearm, the body part making contact on top could be either arm or palm, and movement could either occur from the top body part or the bottom body part. All participants completed touch with these factors.

As we were primarily interested in following up on experiment 1, we focused on palm-on-forearm and forearm-on-palm (henceforth referred to as “models”), these are the only ones included in the main manuscript (fig 2). Within subjects, experiment 2 followed a 2x2 design, with factors top body part (forearm/palm) and movement (top / bottom). In addition, there was a between-subject factor: Half of the participants touched their forearm, and the other half touched their palm (n=15 per group). A full 2x2x2 linear mixed model with all site and movement combinations (delivering site x receiving site x movement) is available in the supplements (Table S1, fig S1).

#### 3.2.3 Procedure

Preceding the experimental trials, each participant was trained to follow a touch protocol. Specifically, participants were asked to practice gentle stroking of their forearm or palm at 3 cm/s (i.e. CT optimal speed) in a distal to proximal direction. Stroking velocity was measured using a digital timer. One practice trial of each condition was completed without any ratings.

Touch occurred in blocks, where each touch type was repeated once per block (randomized within block), across 9 blocks. Following each touch, participants were asked to rate the touch on the three dimensions softness/smoothness/comfort (presentation order randomized for each trial). The ratings were done on a VAS slider, with anchor points being “0” on the far left, and “100” on the far right. Ratings were instructed as 0 being “not soft/smooth/pleasant” and 100 being “extremely soft/smooth/pleasant”, rather than the preferences of self/other used in experiment 1.

#### 3.2.4 Data analysis

The VAS ratings were converted into values from 0 to The average rating for each touch type was calculated for each dimension. Linear mixed models (Satterthwaite method) with fixed effects model (palm-on-forearm / forearm-on-palm) and movement (top / bottom), with random effects subject were done for all dimensions. Secondary analysis including all touch interactions is available in the supplements. Fixed effects estimates were calculated for each factor and interaction. The formula as such read **touch rating ~ model + movement + (1** | **subject)**. Standardized fixed-effect estimates are reported as well as effect sizes Cohen’s f^2^.

### 3.3 Results

#### 3.3.1 Softness

**Figure 3:**
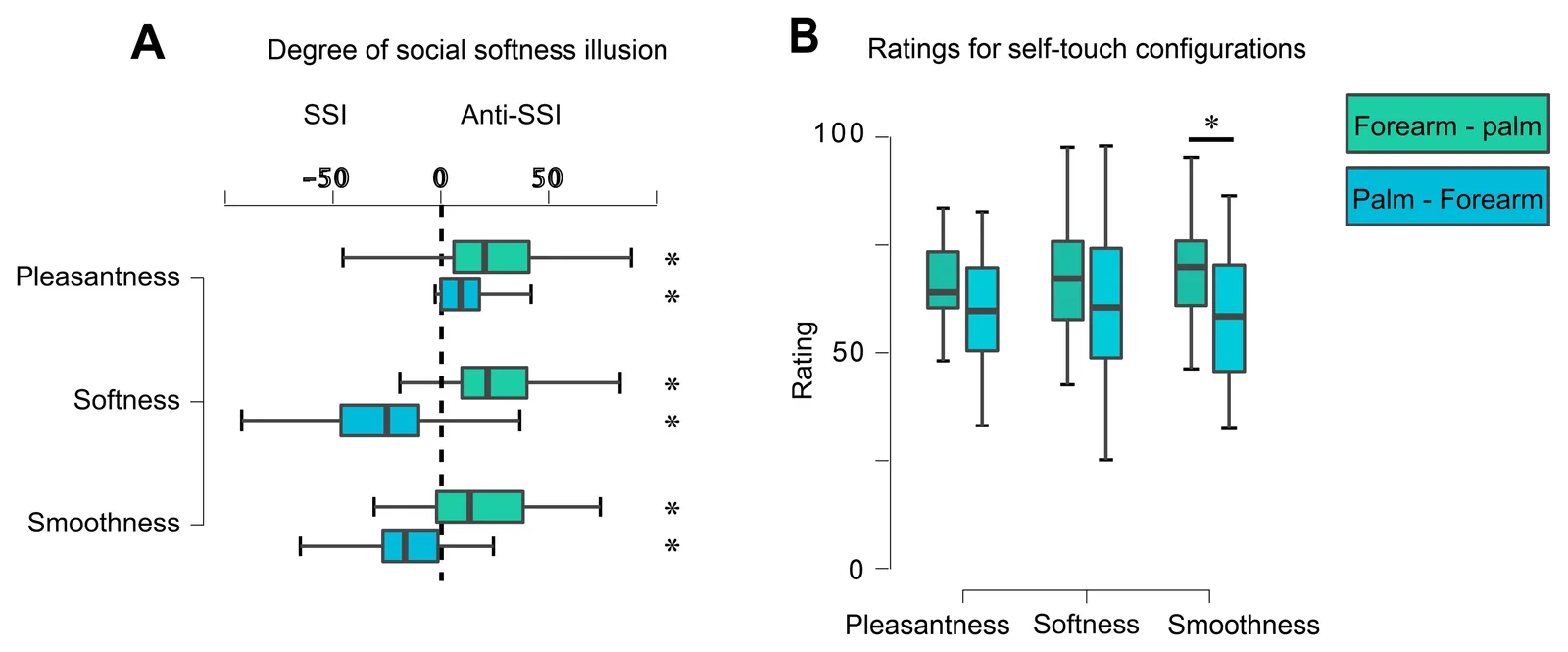
A) Degree of social softness illusion in experiment 1. Preferences for the three dimensions (pleasantness, softness, and smoothness). All were tested with a t-test against 0 (no preference). *Significantly different from 0 after Bonferroni-Holms corrections for multiple comparisons. B) Ratings of self-touch across the three dimensions for touch interactions forearm-palm and palm-forearm *Significant difference between touch configurations. Error bars show highest and lowest values not classified as outliers (i.e. not exceeding 1.5 IQR).

A main effect of movement was found, F (1, 28.0) = 13.033, p = 0.001, fixed effect estimate = 3.037, f^2^ = 0.318, with higher ratings when movement was done by the top body part. No main effect of model was found, F (1, 28.0) = 1.088, p = 0.306, fixed effect estimate = 3.322, f^2^ = 0.037, fig 3b, nor a significant interaction between movement and model, F (1, 28.0) = 0.770, p = 0.388, fixed effect estimate = 0.738, f^2^ = 0.027.

#### 3.3.2 Smoothness

A main effect of movement was found, F (1, 28.0) = 5.927, p = 0.022, fixed effect estimate = 2.275, f^2^ = 0.175 with higher ratings when movement was done by the top body part. A main effect of model was also found, F (1, 28.0) = 7.787, p = 0.009, f^2^ = 0.218 fixed effect estimate = 6.625, with higher ratings for the set up “forearm on top, palm on bottom” compared to “palm on top, forearm on bottom”, fig 3b. The interaction movement by model was not significant, F (1, 28) = 3.473, p = 0.073, fixed effect estimate = 1.742 f^2^ = 0.110.

#### 3.3.3 Pleasantness

A main effect of movement was found, F (1, 28.0) = 6.165, p = 0.019, fixed effect estimate = 2.522, f^2^ = 0.180, with higher ratings when movement was done by the top body part. No main effect of model was found, F (1, 28.0) = 2.790, p = 0.106, fixed effect estimate = 3.605, f^2^ = 0.091, fig 3b. The interaction movement by model was not significant, F (1, 28.0) = 0.559, p = 0.461, fixed effect estimate = 0.760, f^2^ = 0.020.

### 3.4 Interim Discussion Experiment 2

In experiment 2, we looked at how the dual touch occurring during self-touch is affected by body part position and movement. In line with our hypothesis, we found forearm-on-palm was rated as significantly smoother than palm-on-forearm. Softness and pleasantness were descriptively similar but did not reach the significance threshold. Conceptually, these models should be the same, as comparable tactile information is provided in both conditions. Therefore, when investigating self-touch, the interaction between delivering and receiving body part must be considered. Few studies have explicitly asked how touching and touched components interact in self-touch; most studies instead focus on how the touched site differs between self-touch and other touch (S.-J. Blakemore et al., 1999; Kilteni & Ehrsson, 2020; Shergill et al., 2005). Further, these studies mainly assess intensity rather than qualitative aspects of touch. Intensity ratings have been found to be higher for the body part receiving touch compared to the body part delivering the touch. However, intensity does not necessarily translate to the qualitative aspects studied in the current experiments.

Contrary to our hypothesis on movement, we did not observe any interaction between movement (top/bottom) and model. We instead found a main effect of movement: When the top body part was moving, ratings increased for all subscales (softness / smoothness / pleasantness) for both models. This is corroborated in the supplement: independently of the body site delivering or receiving touch, ratings were always higher when movement was done by the top body part. It is possible that movement with the top body part was more predictable due to familiarity. Indeed, predictability has been suggested as one of the primary mechanisms underlying the sensory attenuation observed during self-touch (S.-J. Blakemore et al., 1999; Boehme, 2025). Additional support is provided by studies showing attenuation following imagined movement (Kilteni et al., 2018) or the attribution of movement (Burin et al., 2017). As a counter-argument to this, our sample was primarily right-handed, and self-touch seems to occur mainly by the non-dominant hand (Zhang et al., 2020). While motor tasks differ between dominant and non-dominant hand (A. T. Özcan et al., 2004) tactile acuity does not seem differ (A. Özcan et al., 2005). Again, these studies primarily assess intensity or tactile acuity, and the influence of predictability on qualitative aspects of touch remains elusive.

## 4 Experiment 3

### 4.1 Problem Formulation

Experiment 3 aimed to examine the role of movement, particularly how active and passive movement affects touch perception. The sensorimotor predictions strongly influence the touch sensation (S. J. Blakemore et al., 1998). The SSI has been found to be stronger during active touch (Gentsch et al., 2015), and self-touch attenuation has been found to depend on active rather than passive movements (Kilteni & Ehrsson, 2020). There is strong bidirectional influence between the motor information and tactile senses for spatial estimation, which is particularly pronounced during active movements, rather than during passive movement (Cataldo et al., 2022). However, whether qualitative aspects of touch are experienced differently during active and passive movement remains unclear.

For experiment 3, the dimension of smoothness was replaced by arousal, as smoothness and softness strongly correlated in experiment 2 (supplement). We hypothesized that the slow velocity (3 cm/s) would be rated as more pleasant and softer than the fast velocity (18 cm/s). We also hypothesized a velocity-by-body site interaction, with higher ratings of pleasantness and softness for the forearm at slow velocity, given higher innervation of CT-afferents in the hairy skin. Finally, and most importantly, we expected the higher predictability of active movements to be associated with an increase in subjective ratings for the three dimensions.

### 4.2 Methods

#### 4.2.1 Participants

The same thirty participants as in experiment 2 were included in experiment 3.

#### 4.2.2 Setup

**Figure 4:**
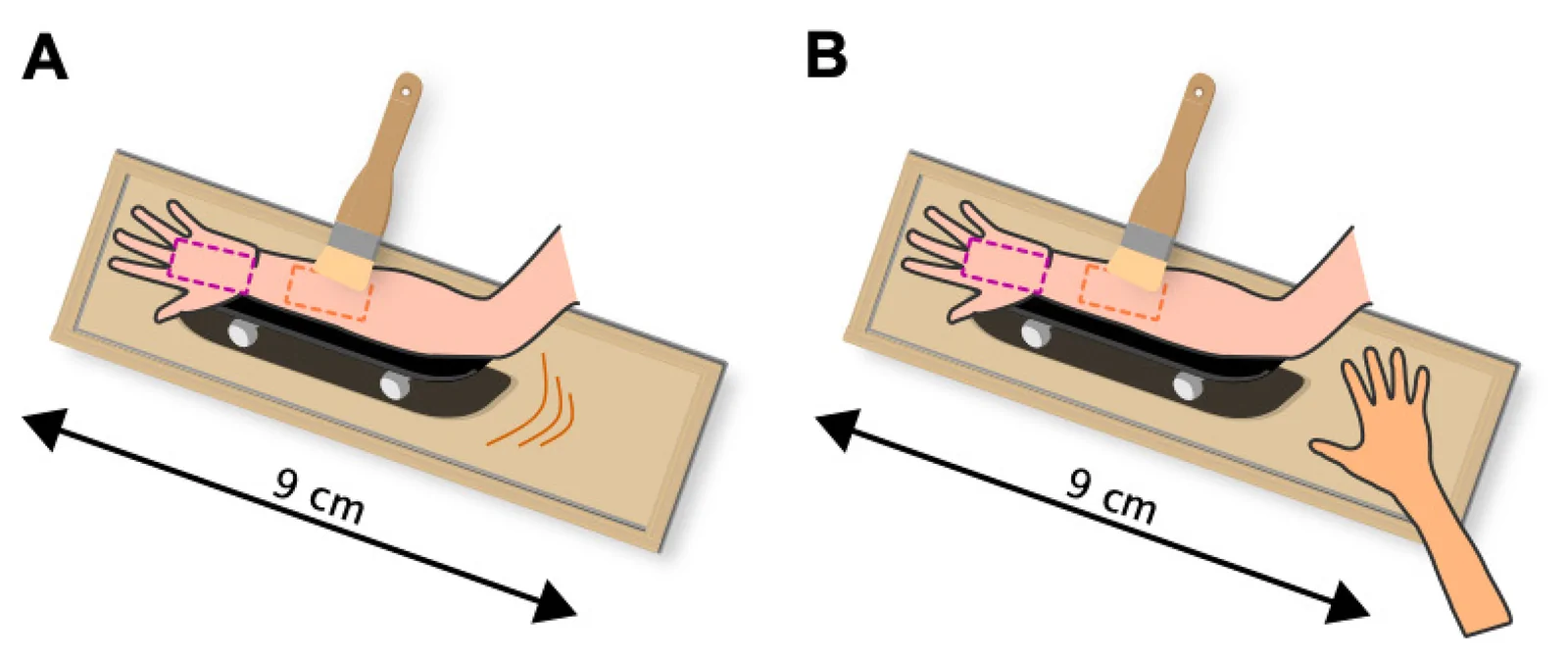
Setup experiment 3. Participants’ left arm was placed on a platform and a makeup brush was placed above it, stroking the volar forearm during movement. Movements were 3 cm/s and 18 cm/s. Touch occurred on one of two sites, palm (purple box) or forearm (orange box), randomized between participants. Movement could either be A) Self-initiated, or B) generated by the experimenter.

As in experiment 2, participants were seated in front of a computer screen with their left arm placed on a moveable platform, with the palm facing upwards. The platform was again placed in a wooden frame allowing it to move 9 cm back and forth but preventing sideways movement. A 9x4 cm area marked was marked in the proximal-distal orientation along their left forearm or palm (n = 15 per area). A makeup brush (Natural hair Blush Brush, N°7, The Boots Company) was positioned above the participants’ arm, stroking the skin when the platform was moving. Movements could either be active and executed by the participant (fig 4a), or passive and executed by the experimenter (fig 4b). The setup is shown in figure 3. Two velocities were used, 3 cm/s and 18 cm/s, to investigate how velocity modulates the experience, providing insight into a potential involvement of CT-afferents (Löken et al., 2009). The movements were timed to ensure the correct velocity was achieved for each trial.

Experiment 3 was a 2×2 design within subjects, with factors velocity (3 / 18 cm/s) and movement (active / passive). There was an additional between subject factor: Half of the participants (n=15 per group) had their forearm stroked by the brush, and the other half has their palm stroked.

#### 4.2.3 Procedure

As in experiment 2, touch occurred in blocks. Each block consisted of four touch types in a 2-by-2 design. Participants either moved the skateboard (active, fig. 4a) or the experimenter would push the skateboard (passive, fig 4b). The velocity of movement could be 3 cm/s or 18 cm/s. Instructions on condition type were provided on the monitor in front of the participant. Each touch occurred once per block, randomized order within blocks, across 9 blocks. After each touch trial, participants rated the trial across three dimensions (Arousal/Pleasantness/Softness; presentation order randomized within each trial) on a VAS scale presented on the monitor, with the far left displaying 0, representing “Not at all arousing/pleasant/soft” and the far right displaying 100, representing “extremely arousing/ pleasant/soft”.

#### 4.2.4 Data analysis

The VAS ratings were converted into values ranging from 0 to 100. The average rating for each touch type was calculated per dimension. A 2-by-2 repeated-measures ANOVA was done for each of the three dimensions with fixed effects Movement and Velocity. Body part (palm / forearm) was used a between subject factor Partial eta squared are reported for each effect. Effect sizes are reported using partialeta squared (η_p_^2^)

### 4.3 Results

#### 4.3.1 Softness

**Figure 5:**
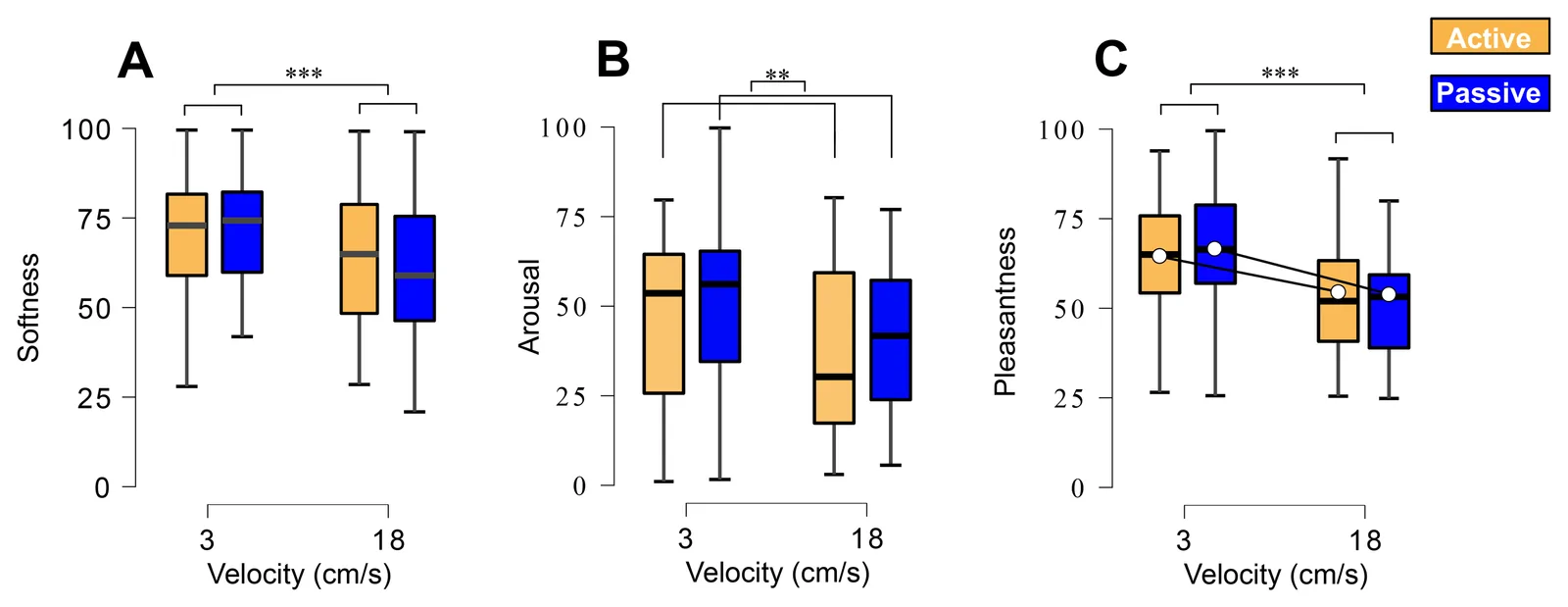
Ratings for our two velocities by movement type, active or passive. Error bars show highest and lowest values not classified as outliers (i.e. not exceeding 1.5 IQR). A) Softness ratings significantly differed between the velocities, with higher ratings for the slow velocity, but no difference by movement type. B) Arousal differed by both velocity and movement type, with higher arousal for passive movements and the slower velocity. C) The slow velocity was perceived as more pleasant. Further, velocity by movement type interacted, with a larger effect of velocity during passive movements. The white dots indicate mean value with lines illustrating the direction of the interaction.

The slower velocity of 3cm/s was perceived as more soft: main effect of velocity, F (1, 28) = 16.337, p < 0.001, η_p_^2^ = 0.368, with higher perceived softness during the slow velocity, fig 5a. No other main effect or interaction was significant: movement, F (1, 28) = 0.003, p = 0.909, η_p_^2^ = < 0.001; body part, F (1, 28) = 0.112, p = 0.741, η_p_^2^ = 0.004; velocity-by-body part, F (1, 28) = 0.003, p = 0.957 η_p_^2^ < 0.001; movement-by-body part, F (1, 28) = 2.373, p = 0.135 η_p_^2^ = 0.078; movement-by-velocity, F (1, 28) = 1.110, p = 0.301, η_p_^2^ = 0.038; movement-by-velocity-by-body part, F (1, 28) = 0.305, p = 0.585, η_p_^2^ = 0.011.

#### 4.3.2 Arousal

The slower velocity of 3cm/s was also perceived as more arousing: main effect of velocity, F (1, 28) = 14.445, p < 0.001, η_p_^2^ = 0.340, with higher arousal ratings for the slow velocity. Furthermore, passive movement led to higher arousal ratings: main effect of movement, F (1, 28) = 11.911, p = 0.002, η_p_^2^ = 0.298, with higher ratings when movement was passive rather than active (Fig. 5b). No other main effect or interaction was significant: body part, F (1,28) = 1.459, p = 0.237, η_p_^2^ = 0.050; velocity-by-body part, F (1, 28) = 1.061, p = 0.312, η_p_^2^ = 0.037; movement-by-body part, F (1, 28) = 0.431, p = 0.517, η_p_^2^ = 0.015; velocity-by-movement, F (1, 28) = 0.453, p = 0.506, η_p_^2^ = 0.016; velocity-by-movement-by-body part, F (1, 28) = 0.239, p = 0.628, η_p_^2^ = 0.008.

#### 4.3.3 Pleasantness

The slower velocity of 3cm/s was perceived as more pleasant: main effect of velocity, F (1, 28) = 18.819, p < 0.001, η_p_^2^ = 0.402, with higher ratings for the slow velocity. There was also a significant interaction of velocity with movement, F (1, 28) = 8.605, p = 0.007, η_p_^2^ = 0.235, where ratings differed more between the velocities when the movement was passive, and less when movement was active (fig. 5c). No other main effects or interactions were found: movement, F (1, 28) = 0.027, p = 0.870, η_p_^2^ < 0.001; body part, F (1, 28) = 0.002, p = 0.968, η_p_^2^ < 0.001; velocity-by-body part, F (1, 28) = 0.444, p = 0.511, η_p_^2^ = 0.016; movement-by-body part, F (1, 28) = 0.723, p = 0.402, η_p_^2^ = 0.025; velocity-by-movement-by-body part, F (1, 28) = 1.187, p = 0.285, η_p_^2^ = 0.041.

### 4.4 Interim Discussion Experiment 3

In experiment 3, we examined the interaction between active and passive movement and velocity on touch experience. In line with our hypothesis, we found that velocity influenced softness, arousal, and pleasantness. We noted significantly higher ratings for all three dimensions in our slow condition (3 cm/s) relative to our fast condition (18 cm/s). This is in line with previous work, where velocities around 3 cm/s are typically perceived as the most pleasant (Löken et al., 2009; Pawling et al., 2017). We did not find a main effect or interaction with body part for any of the ratings. Previous work is rather heterogenous. Typically pleasantness is found to be rated higher on hairy skin (in the present study corresponding to forearm) compared to glabrous skin (here palm) (Ackerley, Carlsson, et al., 2014; McGlone et al., 2012), however other studies find minimal differences (Crucianelli et al., 2022; Cruciani et al., 2021; Löken et al., 2011). Hairy skin has been shown to be more sensitive to velocity changes, however the perceived pleasantness is modulated by velocity on glabrous skin as well, albeit to a lesser extent (Ackerley, Carlsson, et al., 2014). These studies typically keep the participant stationary and move the brush, whereas the present work also included a movement. Tactile gating during movement is known to influence tactile sensitivity (Kilteni & Ehrsson, 2022), but its effects on pleasantness and other qualitative aspects are not well known. As such, the observed differences could stem from the arm moving, regardless of active or passive. For arousal ratings, we found an effect of velocity, with higher ratings for our slower velocity. Previous work on arousal and velocity is more heterogenous compared to pleasantness by velocity. Studies have found slow (3 cm/s) touch more arousing than fast (18 cm/s) touch (Kirsch et al., 2018), but also arousal to be positively correlated with velocity – as the velocity increases, arousal follows (Xu et al., 2022). Physiological measurements of arousal do typically not show the same effect (Pawling et al., 2017). This indicates that the subjective evaluation of arousal does not correspond to physiological measures of it, and the question arises what the participant is rating when asked about arousal.

Active vs. passive movement showed a significant main effect only for arousal, with higher arousal ratings when the movement was passive. Notably, we found an interaction between velocity and movement in the pleasantness ratings – it seems pleasantness is more affected by velocity when someone else causes the movement, and is less affected when the movement is self-evoked. This is in line with our interpretation of movement in experiment 2, where higher predictability was associated with higher ratings. During movement, tactile gating lowers the tactile acuity (Chapman, 1994) – however these effects are comparable between active and passive movement (Rushton et al., 1981). It is therefore likely that the differences noted between active and passive movement are not driven by gating. Actively moving over a surface with the hand, or the hand remaining passive as the surface moved over it did not influence roughness perception (Lederman, 1981), however different neural substrates underlie active and passive somatosensation (Simões-Franklin et al., 2011), not limited to motor cortex associated with movements.

It is possible the differences observed in experiment 3 are due to the agency of movements. Agency is typically defined as the subjective feeling of causing an outcome (Haggard, 2017), which in experiment 3 corresponds to causing the movement. While agency and movement are typically connected, they are distinct phenomena and one can experience one without the other (Farrer & Frith, 2002; Frith, 2005). For self-touch specifically, agency seems to be driving the attenuation, rather than the movement itself. While experiment 3 did not employ self-touch, but rather touch to the self, the same implications are likely.

## 5 Experiment 4

### 5.1 Problem Formulation

The aim of experiment 4 was to try and disentangle agency and movement, given that the differences in active and passive movements observed in experiment 3 could be driven by an agency effect. To further explore how movement, agency, and velocity interact, as seen in experiment 3, no velocity was instructed, to enable more variability in velocities. Either the rating participant or the collaborator decided the velocity, depending on the conditions. Based on experiment 3, we expected agency, rather than the movement itself, to influence touch perception. We expected these effects particularly for pleasantness, but also for arousal and softness. We further expected ratings to be more influenced by velocity when no agency was present, mainly for pleasantness.

#### 5.2.1 Participants

A new sample of twenty-two naïve female participants were enrolled in experiment 4, nineteen of which were rating participants. The same inclusion and exclusion criteria used in experiment 1 were used. The sample size was limited by procedural constraints on data collection. Participants were assigned into either the rating role (i.e. being touched) or collaborator role (see below). Participants partook as both rater and collaborator, however not within the same session or pair. All participants provided written consent prior to experiment start. All nineteen rating participants completed touch ratings prior to being the collaborator.

#### 5.2.2 Setup

The setup was identical to the one used in experiment 3: participants were seated in front of a computer screen with their left forearm placed on the moveable platform within the 9cm frame. The collaborator participant was seated next to the rating participant. A camera next to the skateboard was used to record the movement, to calculate velocity. No instructions for velocity were given, the participants (both rating and collaborating) instead chose the velocity. In order to separate movement from agency, a 2-by-2 design was employed, with movement and agency (over velocity) as factors. The conditions were: rating participant decides the velocity and moves her forearm; rating participant decides the velocity but collaborating participant pushes skateboard; collaborating participant determines velocity and rating participant moves her forearm; collaborating participant decides velocity and pushes skateboard. The conditions are illustrated in Table 1.

#### 5.2.3 Procedure

Experiment 4 followed the same procedure as experiment 3, however instead of the experimenter, the collaborator pushed the skateboard. Velocities were determined by the participants rather than being fixed. A block design was used, with each condition repeated once within each block (in a randomized order). After each touch occurred, the three dimensions (softness, arousal, and pleasantness) were rated (presentation order randomized). A total of 9 blocks were completed. Upon completion of these blocks, participants underwent a control run. During the control, the experimenter pushed the skateboard at 3 cm/s (fixed velocity) and the participant rated the touch. This was repeated three times, once per dimension (supplement).

#### 5.2.4 Data analysis

The VAS scoring was converted into values ranging from 0 to 100. The average rating for each touch type was calculated for each dimension. Trial velocity was extracted from the video recording, and a condition by dimension mean was calculated. A linear mixed model with fixed effects agency and movement was done for velocity to ensure comparable velocities between conditions (supplement). A linear mixed model with fixed effects velocity, agency, and movement was done for each dimension. Standardized fixed-effect estimates and Cohen’s f^2^ are reported.

### 5.3 Results

#### 5.3.2 Softness

The touch was perceived as softer, when the participant got to decide the velocity herself: main effect of agency, F (1, 51.85) = 8.267, p = 0.006, fixed effect estimate = -7.049, f^2^ = 0.138 with higher ratings for agency (+) compared to no agency (-). Velocity as a main effect did not reach the significance threshold, F (1, 66.43) = 3.621, p = 0.061, fixed effect estimate = 3.786, f^2^ = 0.052, nor did movement, F (1, 51.32) = 0.815, p = 0.371, fixed effect estimate = -2.199, f^2^ = 0.016.

**Figure 6:**
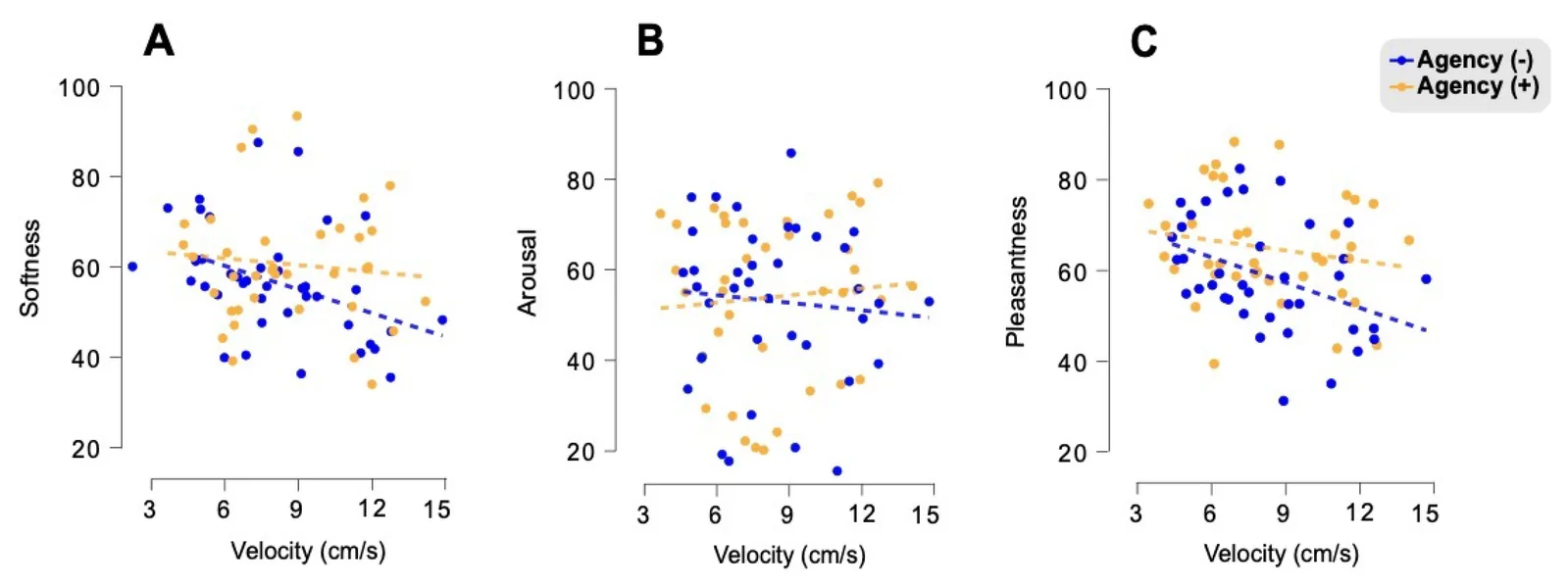
Results experiment 4. Ratings by velocity, split by agency (agency (-) in blue, agency (+) in yellow). A) Softness ratings (interaction significant). B) Arousal ratings (interaction not significant). C) Pleasantness ratings (interaction not significant).

The interaction velocity-by-agency was significant, F (1, 52.05) = 4.255, p = 0.044, fixed effect estimate = 2.009, f^2^ = 0.076 such that when having no agency the effect of velocity on softness rating was larger compared to when having agency (fig. 6a). No other interaction was significant: velocity-by-movement, F (1. 51.29) = 0.565, p = 0.456, fixed effect estimate = 0.725, f^2^ = 0.011; movement-by-agency, F (1, 50.77) = 1.362, p = 0.249, fixed effect estimate = 2.790, f^2^ = 0.026; Velocity-by-agency-by-movement, F (1, 50.81) = 0.569, p = 0.454, fixed effect estimate = -0.714, f^2^ = 0.011.

#### 5.3.3 Arousal

There was no significant effect on arousal ratings (no main effects for velocity, F (1, 67.90) = 3.318, p = 0.079, fixed effect estimate = -4.664, f^2^ = 0.047, agency, F (1, 50.43) = 0.740, p = 0.394, fixed effect estimate = -2.622, f^2^ = 0.014, movement, F (1, 49.98) = 0.008, p = 0.927, fixed effect estimate =-0.278, f^2^ = 0.018 no interaction: velocity-by-agency, F (1, 50.60) = 0.550, p = 0.462, fixed effect estimate = 0.898, f^2^ = 0.011, fig. 6b; velocity-by-movement, F (1, 49.93) = 0.293, p = 0.591, fixed effect estimate = 0.640, f^2^ = 0.006; agency-by-movement, F (1, 49.55) = 0.405, p = 0.527, fixed effect estimate = -1.888, f^2^ = 0.008; velocity-by-agency-by-movement, F (1, 49.58) = 0.229, p = 0.634, fixed effect estimate = 0.562, f^2^ = 0.005).

#### 5.3.4 Pleasantness

As for softness, pleasantness was rated higher when the participant had agency: significant main effect, F (1, 52.99) = 7.817, p = 0.007, fixed effect estimate = -9.007, f^2^ = 0.129, with higher ratings for agency (+) than no agency (-). Velocity was just below the significance threshold, F (1, 46.42) = 3.908, p = 0.054, fixed effect estimate = 4.229, f^2^ = 0.078. Movement was not significant, F (1, 52.39) < 0.001, p = 0.977, fixed effect estimate = -0.092, f^2^ < 0.001.

No interaction was significant, velocity-by-agency was narrowly outside the threshold, F (1, 53.31) = 3.788, p = 0.057, fixed effect estimate = 2.488, f^2^ = 0.066 (Fig. 6c); velocity-by-movement, F (1, 52.43) = 0.356, p = 0.553, fixed effect estimate = 0.758, f^2^ = 0.007; agency-by-movement, F (1, 51.33) = 0.140, p = 0.710, fixed effect estimate = 1.182, f^2^ = 0.003; velocity-by-agency-by-movement, F (1, 51.40) = 0.013, p = 0.908, fixed effect estimate = -0.145, f^2^ < 0.001.

### 5.4 Interim Discussion Experiment 4

In experiment 4, we investigated how velocity, movement, and agency (over velocity) interact and influence touch perception.

In line with our hypothesis, we found a main effect of agency for both softness and pleasantness with higher ratings during agency over the velocity compared to when someone else decided the velocity. Surprisingly, we found no effect of agency on arousal. We found no main effect of movement for either dimension, nor interactions between agency and movement. It therefore seems that agency, i.e. deciding the velocity, is more important than executing the movements. As previously discussed in experiment 3, gating likely affects both active and passive movement similarly (Rushton et al., 1981), and the effect of agency is therefore in line with our hypothesis. Touch perception has been found to be influenced by choice, such as the location touched, but also choice over symbolic factors, such as color of a glove (Gorman et al., 2024). These results align with the present work. Neither study quantified the degree of agency, which would be of particular interest to determine its role on touch perception.

For all three dimensions, velocity was just outside of the significance threshold. While not significant, descriptively these align with our results from experiment 3: for all three dimensions ratings trended upwards during slower velocities. This could in part be explained by the narrow range of velocities noted in experiment 4. Previous literature on touch perception by velocity employs velocities ranging from 0.3 to 30 cm/s, a markedly larger range than those noted in the current work. Ratings typically peak at around 3 cm/s in an inverse u-shape (Löken et al., 2009; Pawling et al., 2017; Walker et al., 2022)). Experiment 4 only captured effects for the moderate to fast velocities, as only these were chosen, and shares the directionality typically observed for these velocities.

Of particular interest is the interaction between agency and velocity in the present study. This interaction was significant for softness ratings and narrowly outside the significance threshold for pleasantness. It seems the velocity is more important when someone else decides it.

## 6 General Discussion

In the present experiments, we investigated how touch perception, especially for self-touch, is influenced by body part interaction, movements, and agency across four experiments. We find that the perception of the softness of touch depends on a multitude of factors, including which body part touches where, which of the touching body parts is moving, and whether the movement and velocity is under perceived self-agency or another person’s agency.

In experiment 1, we replicate the original social softness illusion (Gentsch et al., 2015) where participants show preference for the other person’s skin when touching the forearm with the palm. When instead touching the palm with the forearm, the own skin was preferred, a “self-softness illusion”. This means that the SSI is not purely a social effect but dependent on additional factors. When the body parts were matched (forearm-on-forearm or palm-on-palm) there was no preference. This might stem from the dual touch that occurs during self-touch. During interpersonal touch, there is only sensory information either from the touching or touched body part. However, during self-touch, the sensory information is provided from both touching and touched body part simultaneously (Katz & Krueger, 2013). Previous work argues that the self-specific difference (attenuation) originates from the touched rather than touching body part (Enmalm et al., 2025). As such, it is possible that during self-touch, the preferences shift depending on which site is being actively touched, i.e. the perceptions of the moving part might trump the stationary part’s perceptions (we call this here “dual touch hypothesis”). This could explain the preferences observed during forearm-palm and palm-forearm.

To investigate the dual touch hypothesis further, experiment 2 tested how the delivering and receiving body parts interact with movement. Of particular interest were the differences observed between forearm-on-palm and palm-on-forearm, where ratings were higher for forearm-on-palm, in line with experiment 1.

These higher ratings were present for both movement conditions (main effect), indicating that the body parts involved in self-touch must be considered. This supports the dual-touch hypothesis suggested in experiment 1. Surprisingly, experiment 2 showed consistently higher ratings when the top body part was moving, and no interactions with movement. This is contrary to the dual-touch hypothesis (i.e. that the moving body part would perceive the stationary one) and to previous work showing attenuation driven primarily by the receiving body part. Instead, the high predictability of self-touch has been suggested to drive the attenuation (S.-J. Blakemore et al., 1999; Boehme, 2025). Predictability might be higher when the top body part is the moving part, potentially due to familiarity of the motor information and proprioception in that position (Kilteni et al., 2020; Knoetsch & Zimmermann, 2021).

The specific role of the involved body parts or their relationship with movement has not been evaluated in detail. This was examined in experiment 3. We observed no effect of body site, a main effect of velocity, and a role of movement, however the effects were different for the respective subscales. In line with existing literature, experiment 3 showed velocity to influence pleasantness, with higher ratings for CT-optimal velocity compared to non-CT-optimal faster velocities (Löken et al., 2009; Taneja et al., 2021). This preference was present both on hairy and glabrous skin, and no differences or interactions between body parts were found. This is in line with earlier work showing similar response patterns for both palm and forearm (Crucianelli et al., 2022; Löken et al., 2011; McGlone et al., 2012). Some discrepancies with regard to qualitative aspects of touch for glabrous (palm) compared to hairy (forearm) skin have been observed, where emotional attributes were slightly elevated for hairy skin, and sensory attributes were enhanced for the palm, potentially reflecting underlying innervations (McGlone et al., 2012). This has been replicated, with similar results to the ones reported earlier, i.e. more affective experiences for the forearm compared to the palm (Ackerley, Saar, et al., 2014). Together, these findings highlight the complex interplay between top-down factors influencing touch and potential underlying neurophysiology and assumed importance of CT afferents.

Interestingly, experiment 3 also showed an interaction between the origin of the movement (self vs. other) and velocity – pleasantness was influenced more strongly by velocity when the movement was passive. Higher positive affective values have been observed when receiving touch from a brush rather stroking the own skin with the brush (Ackerley, Saar, et al., 2014). Previous literature has shown that receiving social touch is more pleasant than self-touch (Enmalm et al., 2025). One study found that actively stroking the own arm or palm with a brush evoked a larger response in contralateral somatosensory cortex relative to someone else stroking. However, it remains unclear how these differences related to movements, as there was no movement control (Ackerley et al., 2012). The finding is in sharp contrast to other neuroimaging studies on self-touch, that show an attenuated neural response coupled to the attenuated behavioral percept (Boehme et al., 2019; Kilteni & Ehrsson, 2020). However, these studies use either force-matching paradigms (Kilteni & Ehrsson, 2020; Shergill et al., 2005) or skin-to-skin contact (Boehme et al., 2019; Frost-Karlsson et al., 2022; Kaldewaij et al., 2024) rather than a brush stroke to the self. Neither of these assessed pleasantness, and given experimental constraints (such as tapping in force-matching paradigms) rarely consider the effect of velocity.

Previous research in the self-touch attenuation field suggests that agency is more important than the actual movement (Burin et al., 2018; Kilteni et al., 2018, 2020; Pyasik et al., 2019). Therefore, we tried to disentangle these two aspects in experiment 4, where movement was active or passive, and the agency, i.e. control over velocity, could either lie with the participant or a collaborator. In line with our hypothesis, we found that agency, rather than movements, affected the experience. This was the case for both softness and pleasantness, where higher ratings were observed in trials of self-agency over the velocity. No effect was observed for arousal. Clinical conditions such as functional movement disorders support the distinction of agency from movement, both active and passive (Macerollo et al., 2015). Few studies have directly investigated the connection between agency and velocity for sensory experiences, however both are connected to self-perception (mostly during the rubber hand illusion (Crucianelli et al., 2018)) and contribute to embodiment.

The high degree of predictability is suggested as one of the primary mechanisms underlying the attenuation of self-generated sensations (Bays et al., 2006; Harrison et al., 2021). The current work shows a different effect on qualitative dimensions of touch perception rather than those typically observed for intensity. The phenomenology of softness and smoothness could relate differently to the attenuation of the touch sensation, i.e. it is possible that higher predictability enhances the perception of softness of smoothness. This would however not explain the social softness illusion, where another person’s skin is perceived as softer (which is less predictable). The enhanced softness perception may have developed evolutionarily and could be driven by the biological functions of the touch behaviors: a preference towards social touch (palm-forearm) serves as a facilitator of social touch interactions and non-verbal communication (Dunbar, 1993; McIntyre et al., 2022). Similarly, the preference towards the self (forearm-palm) could be related to the role of self-touch as a potential soothing mechanism proposed elsewhere (Boehme & Olausson, 2022; Moszkowski & Stack, 2007). Social and self-touch provide hedonic benefits for different reasons that may drive the preferences for the touch-specific configuration, depending on both the (social) context and underlying neurophysiology (Gentsch et al., 2015).

The current studies are limited by the sample sizes. We only included females to exclude potential interactions of gender between participants/experimenters, which is known to influence touch processing (Gazzola et al., 2012; Russo et al., 2020). It is therefore not clear how the current work translates to other parts of the population. The current experiments are also limited by their reliance on purely self-rated measurements. Testing qualitative aspects of touch is not typically done with psychophysics but could offer interesting insights. Neuroimaging could also be employed to understand the underlying neural systems at work, as both bottom-up sensory information and top-down modulations of self- and social touch are involved. Another limiting factor is the usage of different stimulating media. In experiment 1 and 2, skin-to-skin touch was employed, whereas experiment 3 and 4 instead relied on a brush stimulating the skin. To investigate qualitative aspects of self-touch, skin-to-skin contact is primarily used (see for example the original SSI-investigation by Gentsch et al. (2015) or Enmalm et al. (2025)). Contrary to the skin-to-skin contact, soft brushes are commonly employed when investigating the role of velocity during affective touch (Ackerley, Carlsson, et al., 2014; Crucianelli et al., 2022; Löken et al., 2011). That is, both are commonly used, however the transferability of results between the methods has not been systematically assessed.

## 7 Conclusion

The perceptual qualities of touch sensations are influenced by the interaction of a multitude of factors. While this might be explained by the predictability of the specific configuration of the touch interaction, some contradictions remain with regards to the perception of softness and smoothness. When investigating different types of touch, we find the more predictable configuration to be perceived as more soft/smooth, potentially relating to the suggested self-soothing function of self-touch. However, when a social touch condition was involved, this less predictable condition evoked the softer sensations and was more sensitive to properties such as velocity. This might be driven by an evolutionarily developed specific superiority of social touch perceptions due to their importance for individuals’ survival.

## Supplementary Material

Supplementary Methods

Supplementary Peer Review

## Figures and Tables

**Table 1:** Conditions in experiment 4.

	Collaborator pushes skateboard	Rater moves forearm
Collaborator decides velocity	(-) AGENCY (-) MOVEMENT	(-) AGENCY (+) MOVEMENT
Rater decides velocity	(+) AGENCY (-) MOVEMENT	(+) AGENCY (+) MOVEMENT

## Data Availability

All data and condition averages for each experiment are available at OSF (https://osf.io/3v6bz/).
