# Supplementary material for: Predictability and Agency Shape Self-Touch Perception
Source: Collabra Psychol. Author manuscript; Available in PMC 2026 Nov 13. (PMC7619731; doi:10.1525/collabra.160692)
Supplement: Supplementary file 1 [file EMS217700-supplement-Supplementary_Methods.docx]

**Peer Review and Communication History**

**MS Title**: Predictability and Agency Shape Self-Touch Perception

**Author Names**: Adam Enmalm, Eleonora Vagnoni, Patrick Falk, Aikaterini Fotopoulou, Rebecca Boehme

**Submitted:** Sep 25, 2025

**Editor First Decision**: Revise & Resubmit

Jan 15, 2026

Dear Adam Enmalm,

I have now received two very thorough reviews of your manuscript, “Predictability and agency shape self-touch perception”, from experts in the field. I also independently read the manuscript before consulting these reviews. Both reviewers have evaluated the contribution of this manuscript positively, but have identified a number of distinct issues that will require some revision. I agree with the reviewer points, both the need to discuss the nesting of data within dyads (as is well described by reviewer 1) and the need to clarify some of the methods and possibly tone down claims around mechnism (c-fibres when such transmission is not directly observed) by reviewer 2.

In particular, Reviewer 1’s comments would require re-running the analysis using a multilevel / mixed-model approach to account for the dyad structure. This is a substantial request, but I believe that such modelling will improve the credibility of the findings, so I’d encourage the authors to consider this approach, or provide strong justification in their response if they decline this suggestion. I do believe that this approach feasible within the scope of a revision.

Based on these reviews I think there is promise in the work but that major revisions would be required before the manuscript could be considered for publication. I will leave it up to you whether you decide to undertake these revisions.

If you choose to resubmit, please include a document with a point-by-point response to both the points I list here and the reviewers’ comments, outlining each change made in your manuscript or providing a suitable rebuttal.

Please ensure that your revised files adhere to our author guidelines, and that the files are fully copyedited/proofed prior to upload. Please also ensure that all necessary copyright permissions have been obtained. This may be the last opportunity for major editing, therefore please fully check your file prior to re-submission.

If you have any questions or difficulties during this process, please contact the editorial office at [editorialoffice@collabra.org](mailto:editorialoffice@collabra.org).

If you choose to revise, we ask that you submit your revision within six weeks.

Sincerely,
Norman Farb

## **Reviewer 1**

##### Rating scale questions

|  | Strongly Disagree | Disagree | Neutral | Agree | Strongly Agree |
| --- | --- | --- | --- | --- | --- |
| The study/studies in this manuscript have strong construct validity (good measures and/or manipulations of the constructs the authors wish to study). (Choose “Neutral” if this is not an empirical manuscript) |  |  |  |  | ✔ |
| The study/studies in this manuscript have strong statistical validity (appropriate statistical tests, assumptions are clear and reasonable, no statistical errors, appropriate statistical inferences, etc.). (Choose “Neutral” if this is not an empirical manuscript) |  | ✔ |  |  |  |
| The study/studies in this manuscript have strong internal validity (any causal claims or implications are well-justified, alternative explanations are thoroughly considered, etc.). (Choose “Neutral” if this is not an empirical manuscript, or no causal claims are made or even vaguely implied.) |  |  |  | ✔ |  |
| The study/studies in this manuscript have strong external validity (authors appropriately constrain their conclusions based on the limits of the generalizability of their findings to other contexts (including from lab to real world), other populations, other stimuli or measures, etc.) |  |  |  | ✔ |  |

##### Open response questions

### Please write your review here. The author(s) will see this review. Your identity will not be revealed to the authors unless you also include your name (i.e., sign your review) in this box. It is up to you whether to reveal your identity or not, either is fine.

This manuscript examines how predictability, agency, and body-part configuration shape the perception of self- versus social touch across four experiments. The topic is highly relevant and underexplored, and the multi-experiment approach using several manipulations and rating scales is a major strength.

The study is thoughtfully designed, yet several points require clarification. Testing participants in pairs is perfectly reasonable for the investigation of social touch; however, it remains unclear how this procedure is supposed to “account for variance in skin properties,” as stated in the manuscript. Given the strong influence of social factors on touch perception, it is also important to know whether paired participants knew each other and whether all participants were screened to ensure they were healthy, as familiarity or discomfort could meaningfully affect ratings.

Because participants were tested in dyads, the data are not independent, and this dependency should be handled analytically, for example through multilevel modeling or random effects for dyad. More generally, the statistical reporting lacks sufficient detail. The manuscript should specify which software and statistical toolboxes were used to conduct the analyses and clarify why effect sizes are reported only for Experiments 1 and 3. It is unclear whether the fixed-effect estimates provided for the later experiments are standardized. If they are unstandardized, they cannot be interpreted as effect sizes, which makes it difficult for readers to assess the magnitude and relevance of the reported effects.

A further challenge arises from the fact that conditions differ across experiments—such as using skin-to-skin touch in some and a brush in others, and employing different outcome scales. These differences make it more difficult to compare results directly across the four experiments and should be acknowledged explicitly.

No sample-size justification or power analysis is provided, even though some experiments include relatively small samples (e.g., N = 22) and involve between-subject factors. A sensitivity analysis or rationale would improve the evaluation of the study’s statistical power. Since later hypotheses build on earlier findings, the absence of preregistration should be acknowledged, along with a clear distinction between confirmatory and exploratory analyses.

Further methodological details need clarification. It is not stated whether the order of rating scales (softness, smoothness/arousal, pleasantness) was randomized, which raises the possibility of anchoring effects. The concluding claim that “less predictable sensations were more dependent on known neurophysiological substrates” appears too strong, as no specific fiber pathways were directly tested.

Several smaller issues should also be addressed. The abstract should include sample characteristics and sample sizes, and contains a sentence that needs correction (“The mechanisms underlying these qualitative differences of self- vs social touch for remain unclear”). Figures 3 and 5 should specify the meaning of error bars, and Figure 5C requires clarification of white versus black dots and lines. Some statistical values, such as “F(1,1)” in Experiment 3, appear implausible and should be checked.

Despite these concerns, the manuscript tackles an important question with creativity and methodological breadth. With clearer reporting, improved statistical transparency, and slightly more cautious interpretation, this work has strong potential to make a meaningful contribution to research on affective and social touch. I encourage revision and resubmission.

## **Reviewer 2**

##### Rating scale questions

|  | Strongly Disagree | Disagree | Neutral | Agree | Strongly Agree |
| --- | --- | --- | --- | --- | --- |
| The study/studies in this manuscript have strong construct validity (good measures and/or manipulations of the constructs the authors wish to study). (Choose “Neutral” if this is not an empirical manuscript) |  |  |  | ✔ |  |
| The study/studies in this manuscript have strong statistical validity (appropriate statistical tests, assumptions are clear and reasonable, no statistical errors, appropriate statistical inferences, etc.). (Choose “Neutral” if this is not an empirical manuscript) |  |  |  | ✔ |  |
| The study/studies in this manuscript have strong internal validity (any causal claims or implications are well-justified, alternative explanations are thoroughly considered, etc.). (Choose “Neutral” if this is not an empirical manuscript, or no causal claims are made or even vaguely implied.) |  |  |  | ✔ |  |
| The study/studies in this manuscript have strong external validity (authors appropriately constrain their conclusions based on the limits of the generalizability of their findings to other contexts (including from lab to real world), other populations, other stimuli or measures, etc.) |  |  |  | ✔ |  |

##### Open response questions

### Please write your review here. The author(s) will see this review. Your identity will not be revealed to the authors unless you also include your name (i.e., sign your review) in this box. It is up to you whether to reveal your identity or not, either is fine.

The present manuscript investigates the differences between the input from the skin and how this is interpreted in self-touch and interpersonal touch through a series of four related experiments. Overall, it is interesting and probes questions into self- and other-touch, providing new insights into the complexities of these processes with regard to more emotional perceptual outcomes. I have a number of point that the authors may want to consider.

It is clear that your hypothesis is based upon the activity from CT afferents, yet the paper could be toned-down somewhat, as you do not record from these therefore you cannot postulate directly about the responses from CTs (e.g. end of Introduction). Just because there is a link with firing characteristics of CTs, it does not mean that other mechanoreceptors do not contribute.

Relating to this, you need to address the finding that CTs have been found in the glabrous skin of the hand (Watkins et al., 2021, J Neurophysiol), rather than stating that CTs do not exist on the palm. Although it seems that the relative number of CTs in the glabrous hand skin is low, this must be considered.

General points - methods

- Did the participants know their experimental pair, was there contact between the participants before the experiment?
- It would be good to specify if the same participants were recruited for Experiments 1 and 2 or if they were all different (as per the text for Experiment 3). Also specify this for Experiment 4.
- Did the participants see the touch?
- Why only add partial eta squared calculations only for Experiment 3? Can you add these to the other experiments?

Experiment 1
Methods: Experiment 1 is difficult to visualize and needs to be much better explained.

- How can both participants be facing opposite directions? From the figure, it looks like they are facing the same way, so more detail could be added.
- Did the active participant touch both their skin and the skin of the other participant simultaneously? This is very difficult to understand here.
- Although a standard contact area was marked out on the skin, did the active participant watch the touch to ensure this?
  Results: In 2.3.3, you say ‘did not survive correction for multiple comparisons’ – I presume the statistics relate to the uncorrected comparison. It would be good to add the corrected comparison.

Experiment 3
At the start of the interim discussion, you mention studies on arm and palm comparisons, but I think you also need to add the papers from Löken et al (2011, Brain Res, Pleasantness of touch in human glabrous and hairy skin…) and Cruciani et al (2021, Neurosci & Biobeh Rev, Pleasantness ratings in response to affective touch across hairy and glabrous skin…). The literature as a whole seems to find only small differences between arm and palm, if any.

General discussion
You mention that few studies have investigated self-touch with brush-stroking or touch to the self using brushes. Here, it seems important to mention the study by Ackerley et al (2014, Front Beh Neuro, Quantifying the sensory and emotional perception of touch…) that did exactly this.

Minor comment

- The paper would benefit from a careful read-though as there are a number of minor spelling/grammatical errors (e.g. end of Introduction).`
- I think you need to fix this reference ‘Rochelle Ackerley et al., 2014’ to ‘Ackerley et al., 2014’.
- Typo general discussion: ‘might stem form’ to ‘might stem from’.

**Author Response**
Mar 13, 2026

**Editor / cover letter**

Dear Adam Enmalm,

I have now received two very thorough reviews of your manuscript, “Predictability and agency shape self-touch perception”, from experts in the field. I also independently read the manuscript before consulting these reviews. Both reviewers have evaluated the contribution of this manuscript positively, but have identified a number of distinct issues that will require some revision. I agree with the reviewer points, both the need to discuss the nesting of data within dyads (as is well described by reviewer 1) and the need to clarify some of the methods and possibly tone down claims around mechnism (c-fibres when such transmission is not directly observed) by reviewer 2.

In particular, Reviewer 1’s comments would require re-running the analysis using a multilevel / mixed-model approach to account for the dyad structure. This is a substantial request, but I believe that such modelling will improve the credibility of the findings, so I’d encourage the authors to consider this approach, or provide strong justification in their response if they decline this suggestion. I do believe that this approach feasible within the scope of a revision.

Based on these reviews I think there is promise in the work but that major revisions would be required before the manuscript could be considered for publication. I will leave it up to you whether you decide to undertake these revisions.

If you choose to resubmit, please include a document with a point-by-point response to both the points I list here and the reviewers’ comments, outlining each change made in your manuscript or providing a suitable rebuttal.

Please ensure that your revised files adhere to our author guidelines, and that the files are fully copyedited/proofed prior to upload. Please also ensure that all necessary copyright permissions have been obtained. This may be the last opportunity for major editing, therefore please fully check your file prior to re-submission.

If you have any questions or difficulties during this process, please contact the editorial office at [editorialoffice@collabra.org](mailto:editorialoffice@collabra.org).

If you choose to revise, we ask that you submit your revision within six weeks.

Sincerely,
Norman Farb

Dear Norman Farb,

Thank you for considering our resubmission of our manuscript “Predictability and agency shape self-touch perception” to Collabra Psychology.

We thank the reviewers for their comments and have adjusted the manuscript accordingly, please see the document “Response to reviewers” for a breakdown of our responses and manuscript adjustments. This includes adjustments, a more cautious framing and effect sizes to all experiments. We have also included a reanalysis of data from experiment 1. This data is now analysed with a linear mixed model including grouping random effect of dyad as per the reviewer suggestion. We have also clarified the methodology, particularly experiment 1, including a new figure.

We believe the manuscript has greatly improved with these changes, and we hope it is to your and the reviewers liking.

Thank you on behalf of all co-authors,

Sincerely

Adam

**Reviewer responses**

**Reviewer 1:**

This manuscript examines how predictability, agency, and body-part configuration shape the perception of self- versus social touch across four experiments. The topic is highly relevant and underexplored, and the multi-experiment approach using several manipulations and rating scales is a major strength.

The study is thoughtfully designed, yet several points require clarification. Testing participants in pairs is perfectly reasonable for the investigation of social touch; however, it remains unclear how this procedure is supposed to “account for variance in skin properties,” as stated in the manuscript. Given the strong influence of social factors on touch perception, it is also important to know whether paired participants knew each other and whether all participants were screened to ensure they were healthy, as familiarity or discomfort could meaningfully affect ratings.

Participants were tested in pairs in order to balance rather than controlling the skin properties across ratings. However, individuals were unfamiliar with each other prior to enrolment in the study. This has now been made clear in the methods section.

“Thirty naïve female participants were recruited for experiment 1 (age = 22.8 ± 3.24, range 19-31). Participants were not familiar with each other prior to study enrollment, and verbal communication between participants was restricted to limit the influence of other social factors.”

Dyad effects have now been considered in the analysis, see next point. The manuscript has been revised throughout to more appropriately address the social factors influencing touch, and the methodological decision should more adequately be justified.

1.0 Introdoction: “It remains unclear how the bottom-up sensory input interacts with potential top-down modulations, predictions, and contextual factors, as these are known to influence the experience of touch (Cascio et al., 2019; Suvilehto et al., 2019).”

6.0 General disdcussion: “This has been replicated, with similar results to the ones reported earlier, i.e. more affective experiences for the forearm compared to the palm (Ackerley et al., 2014c). Together, these findings highlight the complex interplay between top-down factors influencing touch and potential underlying neurophysiology and assumed importance of CT afferents. “

Any skin condition affecting the area was cause for exclusion, as was any psychiatric or neurological disorder. This was determined by … and should

Because participants were tested in dyads, the data are not independent, and this dependency should be handled analytically, for example through multilevel modelling or random effects for dyad.

Data from experiment 1 has now been re-analysed with a linear mixed model including a random effect of pair (dyad). To adequately test our hypotheses (i.e. the presence of a preference, a deviation from 0) post-hoc tests were done for all touch-types. The results remained unchanged, the manuscript has been updated with the new analysis. Here is the new data-analysis section from experiment 1. All results sections are also updated but not included here.

“2.2.4. A linear mixed model with the Satterthwaite method was done for all rating scales. Fixed effects were touch interaction (i.e. palm-palm, palm-forearm, forearm-palm, and forearm-forearm), and random grouping effects were participant and pair (i.e. touching dyad). The formula as such read **rating ~ touch interaction + (1 | dyad) + (1 | subject).** To appropriately test whether participants exhibited a significant preference for either self- or other-touch in each condition, post-hoc marginal means were tested against zero, corrected for multiple comparisons using the Bonferroni-Holms correction. Effect sizes of each model are reported using Cohen’s f^2^ and standardized fixed-effect estimates are reported for the follow-up tests.”

More generally, the statistical reporting lacks sufficient detail. The manuscript should specify which software and statistical toolboxes were used to conduct the analyses and clarify why effect sizes are reported only for Experiments 1 and 3.

All data was analyzed using JASP (JASP TEAM) version 0.19.3. No additional softwares or toolboxes were used for the analysis. This has been clarified in the methods section. Effect sizes have now been added for experiment 1 (re-analysed), experiment 2 and experiment 4.

“2.2.4. Data analysis All data was analyzed using JASP version 0.19.3 (JASP Team).

“Effect sizes of each model are reported using Cohen’s f^2^ and standardized fixed-effect estimates are reported for the follow-up tests.”

3.2.4. “Standardized fixed-effect estimates are reported as well as effect sizes Cohen’s f^2^.”

4.2.4. Effect sizes are reported using partial eta squared (η_p_^2^).

5.2.4. Standardized fixed-effect estimates and Cohen’s f^2^ are reported.

It is unclear whether the fixed-effect estimates provided for the later experiments are standardized. If they are unstandardized, they cannot be interpreted as effect sizes, which makes it difficult for readers to assess the magnitude and relevance of the reported effects.

All fixed-effects estimates are standardized, and the manuscript now states this.

“2.2.4. Effect sizes of each model are reported using Cohen’s f^2^ and standardized fixed-effect estimates are reported for the follow-up tests.”

3.2.4. “Standardized fixed-effect estimates are reported as well as effect sizes Cohen’s f^2^.”

4.2.4. Effect sizes are reported using partial eta squared (η_p_^2^).

5.2.4. Standardized fixed-effect estimates and Cohen’s f^2^ are reported.

A further challenge arises from the fact that conditions differ across experiments—such as using skin-to-skin touch in some and a brush in others, and employing different outcome scales. These differences make it more difficult to compare results directly across the four experiments and should be acknowledged explicitly.

This is indeed an issue, and whenever touch is investigated there is always a balance between methodological control and ecological validity. The concluding discussion now discusses this issue in more detail

6.0 General discussion: “Another limiting factor is the usage of different stimulating media. In experiment 1 and 2, skin-to-skin touch was employed, whereas experiment 3 and 4 instead relied on a brush stimulating the skin. To investigate qualitative aspects of self-touch, skin-to-skin contact is primarily used (see for example the original SSI-investigation by Gentsch et al. (2015) or Enmalm et al. (2025)). Contrary to the skin-to-skin contact, soft brushes are commonly employed when investigating the role of velocity during affective touch (Ackerley et al., 2014b; Crucianelli et al., 2022; Löken et al., 2011). That is, both are commonly used, however the transferability of results between the methods has not been systematically assessed.”

No sample-size justification or power analysis is provided, even though some experiments include relatively small samples (e.g., N = 22) and involve between-subject factors. A sensitivity analysis or rationale would improve the evaluation of the study’s statistical power. Since later hypotheses build on earlier findings, the absence of preregistration should be acknowledged, along with a clear distinction between confirmatory and exploratory analyses.

The sample sizes were based on the original study investigating the social softness illusion (Gentsch et al., 2015), which used similar sample sizes. This sample size is commonly used in this type of study: Ackerley et al., 2014 Front. Behav. Neurosci used a sample size of 34. Pyasik et al., 2022 Psychological Research performed a similar investigation into the social softness illusion and self-other distinction with a n = 24, following a power calculation to find a effect size d = 0.25, with ⍺ = 0.25. We therefore believe our sample size is reasonable.

Experiment 4 had a slightly smaller sample size due to procedural constraints on the data collection. This is now appropriately addressed in the manuscript.

Further methodological details need clarification. It is not stated whether the order of rating scales (softness, smoothness/arousal, pleasantness) was randomized, which raises the possibility of anchoring effects.

These were always randomized within individuals for all experiments. This has been clarified in the manuscript now.

2.2.3. After each pair of touches (i.e. self and other), the active participant provided comparative ratings of the two sites touched across three dimensions: softness, smoothness, and comfort. The presentation order of these ratings was randomized within participants.

3.2.3. Following each touch, participants were asked to rate the touch on the three dimensions softness/smoothness/comfort (presentation order randomized for each trial).

4.2.3. After each touch trial, participants rated the trial across three dimensions (Arousal/Pleasantness/Softness; presentation order randomized within each trial)

5.2.3. After each touch occurred, the three dimensions (softness, arousal, and pleasantness) were rated (presentation order randomized).

The concluding claim that “less predictable sensations were more dependent on known neurophysiological substrates” appears too strong, as no specific fiber pathways were directly tested.

The role of neurophysiology has been toned down in this section, instead ascribing most of the effect to properties we did investigate directly, velocity.

7. However, when a social touch condition was involved, this less predictable condition evoked the softer sensations and was more sensitive to properties such as velocity.

Several smaller issues should also be addressed. The abstract should include sample characteristics and sample sizes, and contains a sentence that needs correction (“The mechanisms underlying these qualitative differences of self- vs social touch for remain unclear”).

The abstract has been adjusted and should now include more sample characteristics, and the incomplete sentence have been corrected.

Abstract: Across four experiments, we explored such qualitative aspects of touch sensation in eighty-two healthy females. We investigated both the social softness illusion and self-touch across multiple body parts.

Figures 3 and 5 should specify the meaning of error bars, and Figure 5C requires clarification of white versus black dots and lines.

Figure 5C has now been updated and the lines/dots are now described in the figure legend. The error bars represent the most extreme value not classified as an outlier, with an outlier being defined as 1.5 times the IQR. This is now addressed in the respective figure legends:

“Fig 3: *Error bars show highest and lowest values not classified as outliers (i.e. not exceeding 1.5 IQR).”*

*Fig 5: Ratings for our two velocities by movement type, active or passive. Error bars show highest and lowest values not classified as outliers (i.e. not exceeding 1.5 IQR). A) Softness ratings significantly differed between the velocities, with higher ratings for the slow velocity, but no difference by movement type. B) Arousal differed by both velocity and movement type, with higher arousal for passive movements and the slower velocity. C) The slow velocity was perceived as more pleasant. Further, velocity by movement type interacted, with a larger effect of velocity during passive movements. The white dots indicate mean value with lines illustrating the direction of the interaction.*

Some statistical values, such as “F(1,1)” in Experiment 3, appear implausible and should be checked. Thank you for highlighting this! This was a human error, and these should now read correctly throughout the section.

Despite these concerns, the manuscript tackles an important question with creativity and methodological breadth. With clearer reporting, improved statistical transparency, and slightly more cautious interpretation, this work has strong potential to make a meaningful contribution to research on affective and social touch. I encourage revision and resubmission.

Thank you for giving your thoughts and providing this feedback. We hope the manuscript now is to your satisfaction!

**Reviewer 2:**

The present manuscript investigates the differences between the input from the skin and how this is interpreted in self-touch and interpersonal touch through a series of four related experiments. Overall, it is interesting and probes questions into self- and other-touch, providing new insights into the complexities of these processes with regard to more emotional perceptual outcomes. I have a number of point that the authors may want to consider.

It is clear that your hypothesis is based upon the activity from CT afferents, yet the paper could be toned-down somewhat, as you do not record from these therefore you cannot postulate directly about the responses from CTs (e.g. end of Introduction). Just because there is a link with firing characteristics of CTs, it does not mean that other mechanoreceptors do not contribute.

There is absolutely involvement of more receptors than just the CTs in the current study, however as you correctly note the goal of these studies was to investigate how neurophysiology and subjective experiences of touch interact. Recording CT-activity with for example microneurography allows us to isolate their activity, however this type of investigation has its own problems regarding ecological validity. It is very hard to investigate a ”natural” interaction. Therefore, we relied largely on known neurophysiological differences of hairy and glabrous skin. Nonetheless, the (potential) role of the CT-afferents has been toned down throughout the work.

Relating to this, you need to address the finding that CTs have been found in the glabrous skin of the hand (Watkins et al., 2021, J Neurophysiol), rather than stating that CTs do not exist on the palm. Although it seems that the relative number of CTs in the glabrous hand skin is low, this must be considered.

The findings of Watkins et al., (2021) are partially problematic, as they 1) have not been replicated by others, and 2) two out of the three microneurography units were in the border region between hairy and glabrous skin. Nonetheless, we cannot rule of a sparse innervation of the glabrous skin, and the manuscript has been adjusted to adress this, and more cautious interpretations are presented.

1. Introduction: Slow conducting C-tactile (CT) receptors located primarily in hairy skin (Olausson et al., 2010) show preferential activation to soft, slow stroking at skin temperature and their activation relates specifically to hedonic aspects of touch (Ackerley et al., 2014a; Löken et al., 2009). Sporadic innervation of CT-afferents has been reported for glabrous skin and the glabrous-hairy skin border at roughly one seventh the innervation density (Watkins et al., 2021), although this has not been replicated.

1. Introduction: Across four experiments, we examined factors known to influence qualitative aspects of touch. We examined if and how the SSI varies across body parts with varying CT-innervations, both regarding the delivering and receiving body parts (experiment 1). We further examined how movements interacted with these body parts to investigate the dual-touch during self-touch (experiment 2). We examined how active and passive movements interacted with velocity in shaping qualitative experiences of soft stroking with a brush (experiment 3). Finally, we assessed whether agency rather than movement influences touch and interacts with velocity to shape the tactile experience (experiment 4).

2.1. Experiment 1 aimed to explore the role of the CT-system in the SSI. Since the SSI showed a similar velocity tuning as the CT-system and occurred only when the target area was heavy in CT-innervation, it was suggested that it might occur specifically when activating CTs in the touch-recipient (Gentsch et al., 2015).

6.0 This preference was present both on hairy and glabrous skin, and no differences or interactions between body parts were found. This is in line with earlier work showing similar response patterns for both palm and forearm (Crucianelli et al., 2022; Löken et al., 2011; McGlone et al., 2012). Some discrepancies with regard to qualitative aspects of touch for glabrous (palm) compared to hairy (forearm) skin have been observed, where emotional attributes were slightly elevated for hairy skin, and sensory attributes were enhanced for the palm, potentially reflecting underlying innervations (McGlone et al., 2012). This has been replicated, with similar results to the ones reported earlier, i.e. more affective experiences for the forearm compared to the palm (Ackerley et al., 2014c). Together, these findings highlight the complex interplay between top-down factors influencing touch and potential underlying neurophysiology and assumed importance of CT afferents.

General points - methods

- Did the participants know their experimental pair, was there contact between the participants before the experiment? Study participants did not know each other prior to enrollment, and no contact prior occured. This has been clarified.

2.2.1. Thirty naïve female participants were recruited for experiment 1 (age = 22.8 ± 3.24, range 19-31). Participants were not familiar with each other prior to study enrollment, and verbal communication between participants was restricted to limit the influence of other social factors. (…) participants were assigned into pairs, alternating between being the active and passive participant.

- It would be good to specify if the same participants were recruited for Experiments 1 and 2 or if they were all different (as per the text for Experiment 3). Also specify this for Experiment 4. All participants were naive (i.e. one subset for experiment 1, one subset for experiment 2 and 3, and one subset for experiment 4). This has been clarified in the manuscript now.

3.2.1. Participants Thirty female participants were recruited (age = 21.2 ±2.7, range 18-28). This sample was independent from experiment 1 and had no prior exposure to any of the tasks.

5.2.1. Participants A new sample of twenty-two naïve female participants were enrolled in experiment 4.

- Did the participants see the touch? Participants were asked to focus on a computer screen placed in front of them, but touch was not actively hidden out of view. As such, peripheral visual input of the touch likely occurred. The method-sections for each experiment now more clearly states this.

3.2.2. Setup Participants were seated in front of a computer screen and asked to fixate on a “+” presented on the screen.

- Why only add partial eta squared calculations only for Experiment 3? Can you add these to the other experiments? Effect sizes have now been added to the other experiments, in experiment 1, 2, and 4, the effect sizes are reported with cohens f^2^ given the usage of linear mixed models. Experiment 3 still uses partial eta squared.

Experiment 1
Methods: Experiment 1 is difficult to visualize and needs to be much better explained. The methods section of experiment 1 has been rewritten and should read more clearly now. We have also included an additional figure, see next point.

2.2.3. Procedure The task was divided into two blocks, the order of which was randomized and counterbalanced across participants. In one block, the active participant applied touch using their fingertips (palm), and in the other, using their volar forearm. This was counterbalanced across participants. Touch occurred to either the palm or forearm, alternating within blocks. The active participant touched either themselves followed by touching the collaborator participant, or in the inverted order (alternating within blocks). This was to avoid any anchoring of preferences. Touch occurred on both sites but were always paired (i.e. comparative ratings were always within location). After each pair of touches (i.e. self and other), the active participant provided comparative ratings of the two sites touched across three dimensions: softness, smoothness, and comfort. The presentation order of these ratings was randomized within participants. Each dimension was rated on a 200mm visual-analog scale (VAS). The anchors were labeled “Other Skin” (left) and “Own Skin” (right), with the midline representing no preference. A total of 8 ratings per dimension were completed per block. Upon completion of the first block, a short break was provided, after which the second block began, mirroring the first in structure but switching the touching body part. As such, four touch “models” occurred, palm-palm, palm-forearm, forearm-palm, and forearm-forearm. Once both blocks were completed, the roles of the participants (active/passive) were swapped, and the entire procedure was repeated.

- How can both participants be facing opposite directions? From the figure, it looks like they are facing the same way, so more detail could be added. The participants sat facing the opposite directions to allow both participants left arms to be placed parallel to one another. A figure has been added which should more clearly illustrate the setup, please see figure 1a. The setup-part of experiment 1 has also been adjusted to explain this better.

2.2.2. To standardize the area of contact, a 9-by-4 cm area was marked on the volar side of each participant’s forearm and palm, both of which were Participants were seated next to each other facing opposite directions (Figure 1A). The active participant, responsible for delivering the touch, was facing a computer screen where task instructions were provided. The passive participant sat left of the active participant, with their back facing the table. The participants were separated by a curtain to minimize visual input. Both participants rested their forearms on the table in a parallel position, angled approximately 45° relative to the active participant. The active participant was instructed to fixate on the computer screen where a “+” was presented.

- Did the active participant touch both their skin and the skin of the other participant simultaneously? This is very difficult to understand here. The active participants touched first their own skin and then the other participant’s skin (or vice-versa). They always took turns touching the two skin sites followed by a rating, and both orders were used (starting order randomized between participants). The methods section explains this more clearly now.

The active participant touched either themselves followed by touching the collaborator participant, or in the inverted order (alternating within blocks).

- Although a standard contact area was marked out on the skin, did the active participant watch the touch to ensure this?
  Results: In 2.3.3, you say ‘did not survive correction for multiple comparisons’ – I presume the statistics relate to the uncorrected comparison. It would be good to add the corrected comparison. Results from study 1 have been re-analysed and p-values now refer to corrected p-values.

Experiment 3
At the start of the interim discussion, you mention studies on arm and palm comparisons, but I think you also need to add the papers from Löken et al (2011, Brain Res, Pleasantness of touch in human glabrous and hairy skin…) and Cruciani et al (2021, Neurosci & Biobeh Rev, Pleasantness ratings in response to affective touch across hairy and glabrous skin…). The literature as a whole seems to find only small differences between arm and palm, if any.

This was more correctly phrased in the main discussion at the end, however the interim discussion of experiment 3 now more accurately discusses its findings and the relation to the papers suggested, which are now cited, and other papers which together show a rather heterogenous effects of skin type on touch pleasantness. The main differences between skin type seems to be velocity-modulation, i.e. the inverted U-shape is deeper in hairy skin, although this has not been systematically addressed.

In experiment 3, we examined the interaction between active and passive movement and velocity on touch experience. In line with our hypothesis, we found that velocity influenced softness, arousal, and pleasantness. We noted significantly higher ratings for all three dimensions in our slow condition (3 cm/s) relative to our fast condition (18 cm/s). This is in line with previous work, where velocities around 3 cm/s are typically perceived as the most pleasant (Löken et al., 2009; Pawling et al., 2017). We did not find a main effect or interaction with body part for any of the ratings. Previous work is rather heterogenous. Typically pleasantness is found to be rated higher on hairy skin (in the present study corresponding to forearm) compared to glabrous skin (here palm) (Ackerley et al., 2014b; McGlone et al., 2012), however other studies find minimal differences (Crucianelli et al., 2022; Cruciani et al., 2021; Löken et al., 2011). Hairy skin has been shown to be more sensitive to velocity changes, however the perceived pleasantness is modulated by velocity on glabrous skin as well, albeit to a lesser extent (Ackerley et al., 2014b). These studies typically keep the participant stationary and move the brush, whereas the present work also included a movement. Tactile gating during movement is known to influence tactile sensitivity (Kilteni & Ehrsson, 2022), but its effects on pleasantness and other qualitative aspects are not well known. As such, the observed differences could stem from the arm moving, regardless of active or passive. For arousal ratings, we found an effect of velocity, with higher ratings for our slower velocity. Previous work on arousal and velocity is more heterogenous compared to pleasantness by velocity. Studies have found slow (3 cm/s) touch more arousing than fast (18 cm/s) touch (Kirsch et al., 2018), but also arousal to be positively correlated with velocity – as the velocity increases, arousal follows (Xu et al., 2022). Physiological measurements of arousal do typically not show the same effect (Pawling et al., 2017). This indicates that the subjective evaluation of arousal does not correspond to physiological measures of it, and the question arises what the participant is rating when asked about arousal.

General discussion
You mention that few studies have investigated self-touch with brush-stroking or touch to the self using brushes. Here, it seems important to mention the study by Ackerley et al (2014, Front Beh Neuro, Quantifying the sensory and emotional perception of touch…) that did exactly this.

Thank you for highlighting this paper. We have now incorporated it into the discussion, and the discussion section dealing with this largely been rewritten to improve clarity.

In line with existing literature, experiment 3 showed velocity to influence pleasantness, with higher ratings for CT-optimal velocity compared to non-CT-optimal faster velocities (Löken et al., 2009; Taneja et al., 2021). This preference was present both on hairy and glabrous skin, and no differences or interactions between body parts were found. This is in line with earlier work showing similar response patterns for both palm and forearm (Crucianelli et al., 2022; Löken et al., 2011; McGlone et al., 2012). Some discrepancies with regard to qualitative aspects of touch for glabrous (palm) compared to hairy (forearm) skin have been observed, where emotional attributes were slightly elevated for hairy skin, and sensory attributes were enhanced for the palm, potentially reflecting underlying innervations (McGlone et al., 2012). This has been replicated, with similar results to the ones reported earlier, i.e. more affective experiences for the forearm compared to the palm (Ackerley et al., 2014c). Together, these findings highlight the complex interplay between top-down factors influencing touch and potential underlying neurophysiology and assumed importance of CT afferents.

Interestingly, experiment 3 also showed an interaction between the origin of the movement (self vs. other) and velocity – pleasantness was influenced more strongly by velocity when the movement was passive. Higher positive affective values have been observed when receiving touch from a brush rather stroking the own skin with the brush (Ackerley et al., 2014c). Previous literature has shown that receiving social touch is more pleasant than self-touch (Enmalm et al., 2025). One study found that actively stroking the own arm or palm with a brush evoked a larger response in contralateral somatosensory cortex relative to someone else stroking. However, it remains unclear how these differences related to movements, as there was no movement control (Ackerley et al., 2012). The finding is in sharp contrast to other neuroimaging studies on self-touch, that show an attenuated neural response coupled to the attenuated behavioral percept (Boehme et al., 2019; Kilteni & Ehrsson, 2020). However, these studies use either force-matching paradigms (Kilteni & Ehrsson, 2020; Shergill et al., 2005) or skin-to-skin contact (Boehme et al., 2019; Frost-Karlsson et al., 2022; Kaldewaij et al., 2024) rather than a brush stroke to the self. Neither of these assessed pleasantness, and given experimental constraints (such as tapping in force-matching paradigms) rarely consider the effect of velocity.

Minor comment

- The paper would benefit from a careful read-though as there are a number of minor spelling/grammatical errors (e.g. end of Introduction).`
- I think you need to fix this reference ‘Rochelle Ackerley et al., 2014’ to ‘Ackerley et al., 2014’. Thank
- Typo general discussion: ‘might stem form’ to ‘might stem from’.

Thank you for highlighting these, these have now all been corrected.

**Editor Final Decision:** Accept

Apr 1, 2026

Dear Dr. Enmalm,

I have now had a chance to read over your manuscript "Predictability and agency shape self-touch perception”, along with the letter describing the changes you made. Thank you for your responsiveness to the concerns that the reviewers and I raised. I am happy to say that your paper is now officially accepted for publication in Collabra: Psychology. Congratulations on this excellent work, I think it will make an important contribution to the literature and I look forward to seeing it published! I hope your experiences with Collabra: Psychology have been positive and that you will continue to consider it as an outlet for your work.

As there are no further reviewer revisions to make, you do not have to complete any tasks at this point.

You will be receiving separate correspondence regarding any production and technical comments, data deposits, as well as publication charges. We work with the Copyright Clearance Center to process any applicable APC charges. Please note that your APC transaction must be completed before your article gets published. Corresponding authors who are active SIPS members are eligible for a discounted APC rate of $975 for papers submitted through June 30, 2026. Our managing editor will be in contact with you in the next few days to address any remaining issues and offer you a SIPS member discount code if you are an active SIPS member.

You will have an opportunity to check the page proofs before we publish your article. Thank you again for publishing in Collabra: Psychology.

Sincerely,
Norman Farb
