# Supplementary material for: Predictability and Agency Shape Self-Touch Perception
Source: Collabra Psychol. Author manuscript; Available in PMC 2026 Nov 13. (PMC7619731; doi:10.1525/collabra.160692)
Supplement: Supplementary file 2 [file EMS217700-supplement-Supplementary_Peer_Review.docx]

**Predictability and Agency Shape Self-Touch Perception—Supplements**

**Experiment 1**

Method: Participants were given the opportunity to practice the touch task prior to starting. This was to keep both velocity and pressure consistent. Velocity was kept at 3 cm/s, which is typically classified as the most pleasant, and CT-optimal (Löken et al., 2009). This was done using a digital clock for timing. Force was kept below 0.3N (i.e. approximately 100g) and practiced using a digital scale, in order to keep the touch gentle. Only after the experimenter considered the participant able to comply with the task did the experiment begin.

Supplementary analysis: A 2-by-2 repeated measures ANOVA was conducted on preferences with factor touching site (palm/forearm) and touched site (palm/forearm) for the three dimensions to investigate effects of body part rather than specific interactions. For softness we found main effects of touching site, F (1, 29) = 19.851, p < 0.001, where when touching with the palm there was a stronger preference to the other person, and when touching with the forearm there was a preference towards self. There was also a main effect of touched site, F (1, 29) = 21.481, p < 0.001: when touched on the forearm there was a preference to the other person; and when touched on the palm there was a preference towards self. The interaction was non-significant, F (1, 29) = 0.025, p = 0.875. For smoothness, the same pattern was observed: A main effects of touching site, F (1, 29) = 16.058, p < 0.001, where the palm there showed preference towards other person, and when touching with the forearm there was a preference towards self. A main effect of touched site, F (1, 29) = 14.443, p < 0.001: when the forearm was touched there was a preference to the other person; and the palm was touched there was a preference towards self. The interaction was again not significant, F (1, 29) = 0.393, p = 0.536. Finally, for comfort there was again a main effect of touching site, F (1, 29) = 6.820, p = 0.014. Both touching sites had a preference towards self, but a stronger preference was noted when touch occurred with the forearm. There was no main effect of touched site, F (1, 29) = 0.512, p = 0.480, nor an interaction, F (1, 29) = 2.122, p = 0.156.

**Experiment 2**

Table S1.

*Conditions of Experiment 2*

| **Condition** | **Body part at**  **Bottom** | **Body Position** | **Body Part on Top** |
| --- | --- | --- | --- |
| 1 | Palm | Top arm moving | Palm |
| 2 | Palm | Top arm moving | Forearm |
| 3 | Palm | Bottom arm moving | Palm |
| 4 | Palm | Bottom arm moving | Forearm |
| 5 | Forearm | Top arm moving | Palm |
| 6 | Forearm | Top arm moving | Forearm |
| 7 | Forearm | Bottom arm moving | Palm |
| 8 | Forearm | Bottom arm moving | Forearm |

Fig S1. Conditions of experiment 2. Within participants the body part on top (palm/forearm) and the origin of the movement (top/bottom) was manipulated. Between subjects, the body part on bottom was either the forearm or palm, shown by the orange and purple dotted lines respectively.

Supplementary analysis: An additional linear mixed model was run to supplement the main analysis. In this model, the fixed effects were top body part (palm/forearm), bottom body part (palm/forearm) and movement (touching/touched).

The linear mixed models for all three dimensions are shown in table S2. For softness, we found a main effect of movement, with higher ratings when the top body part was moving; a main effect of top body part, with higher ratings when the top body part was the forearm. No other significant effects or interactions were noted. For smoothness, the same effects and directionalities were noted. For comfort, movement and body part position (top) were both significant main effects, in line with softness and smoothness. We also found a significant interaction between body part– when touching with the palm, the touched surface mattered more.

| *Table S2.* | | | | | | | | | |
| --- | --- | --- | --- | --- | --- | --- | --- | --- | --- |
|  | **Softness** | | | **Smoothness** | | | **Pleasantness** | | |
| **Effect** | **df** | **F** | **p** | **df** | **F** | **p** | **df** | **F** | **p** |
| Movement | 1, 84.00 | 5.298 | 0.024 | 1, 84.00 | 6.581 | 0.012 | 1, 84.00 | 9.500 | 0.003 |
| Body part on top | 1, 84.00 | 63.226 | < 0.001 | 1, 84.00 | 81.564 | < 0.001 | 1, 84.00 | 29.891 | < 0.001 |
| Body part on bottom | 1, 28.00 | 2.676 | 0.113 | 1, 28.00 | 0.495 | 0.487 | 1, 28.00 | 0.058 | 0.811 |
| Movement ✻ Body part on top | 1, 84.00 | 0.012 | 0.913 | 1, 84.00 | 0.832 | 0.364 | 1, 84.00 | 1.608 | 0.208 |
| Movement ✻ Body part on bottom | 1, 84.00 | 0.423 | 0.517 | 1, 84.00 | 1.010 | 0.318 | 1, 84.00 | 0.068 | 0.795 |
| Body part on top ✻  Body part on bottom | 1, 84.00 | 1.628 | 0.205 | 1, 84.00 | 3.302 | 0.073 | 1, 84.00 | 4.892 | 0.030 |
| Movement ✻ Body part on top ✻ Body part on bottom | 1, 84.00 | 0.679 | 0.412 | 1, 84.00 | 0.004 | 0.952 | 1, 84.00 | 0.069 | 0.794 |

Supplementary discussion Experiment 2: Finally, softness and smoothness were correlated in across all touch conditions. A significant correlation was noted, r = 0.719, p < 0.001, Fisher’s z = 0.907.

**Experiment 3**

Body part: No main effect of body part was found for either subscale: softness, F (1) = 0.112, p = 0.741; arousal, F (1) = 1.459, p = 0.237; pleasantness, F (1) = 0.002, p = 0.968.

**Experiment 4**

Velocity: A Supplementary linear mixed model was done with dependent variable velocity in the participant-collaborator trials. The model contained fixed effects agency (yes/no) and movement (self/other), with participant as random factor.

Mean velocity for all trials was 8.4 cm/s (SD = 2.7 cm/s, range 3.6-14.7 cm/s). The linear mixed model for velocity was done with factors movement (self/other) and agency (yes/no). No main effect of agency was found, F (1, 54.0) = 0.148, p = 0.702, nor a main effect of movement, F (1, 54.0) = 1.261, p = 0.267. The interaction movement-by-agency was not significant, F (1, 54.0) = 0.761, p = 0.387. Together, this indicates no systematic differences in velocity across conditions or dimensions.

Control ratings: A control linear mixed model was done for the control ratings when movement were done yb and velocity was determined by the experimenter (3 cm/s). This model contained fixed effects movement (self/other/experimenter) and velocity, and random effect participant.

Softness: A significant main effect was found for velocity, F (2, 86.93) = 12.125, p < 0.001. A main effect was also found for who performed the movements, F (2, 76.36) = 7.372, p = 0.001. Post-hoc asymptotic z-test Bonferroni-Holm corrections showed a significant difference between other and researcher, z = 3.117, p = 0.005, as well as a significant difference between self and researcher, z = 3.125, p = 0.005, with higher ratings for when the experimenter moved the arm. No difference was found between self- and collaborator movements, z = 0.177, p = 0.860. The interaction velocity-by-movement was also significant, F (2, 77.68) = 5.922, p = 0.004.

Arousal: A significant main effect was found for velocity, F (2, 82.20) = 13.919, p < 0.001. A main effect was also found for who performed the movements, F (2, 73.71) = 6.745, p = 0.002. A significant difference between other and researcher, z = 3.513, p = 0.001, as well as a significant difference between self and researcher, z = 3.553, p = 0.001, was determined using post-hoc asymptotic z-test Bonferroni-Holm corrections. with higher ratings for when the experimenter moved the arm. No difference was found between self- and collaborator movements, z = 1.069, p = 0.285. The interaction velocity-by-movement was also significant, F (2, 77.68) = 5.922, p = 0.004.

Pleasantness: The model showed only a significant effect of velocity, F (2, 88.80) = 5.014, p = 0.028. Movement was not significant, F (2, 77.72) = 2.956, p = 0.058, nor was the interaction velocity by movement, F (2, 79.03) = 2.425, p = 0.095.
